# Multiple distinct domains of human XIST are required to coordinate gene silencing and subsequent heterochromatin formation

**DOI:** 10.1186/s13072-022-00438-7

**Published:** 2022-02-04

**Authors:** Thomas Dixon-McDougall, Carolyn J. Brown

**Affiliations:** Department of Medical Genetics, University of British Columbia, Vancouver, BC Canada

**Keywords:** XIST, Long non-coding RNA, Epigenetics, Heterochromatin, CRISPR, Gene silencing, IF-FISH, X-chromosome inactivation, Dosage compensation

## Abstract

**Background:**

Mammalian dosage compensation is achieved by the inactivation of one X chromosome in XX individuals. In eutheria this process is initiated early in development by the long non-coding RNA XIST. Studies of the initiation of silencing by XIST have focussed on mouse models, so the domains of XIST required to induce silencing in humans, and their relationship with domains required to establish heterochromatin remain to be determined.

**Methods:**

We have previously established an inducible XIST cDNA in somatic cells and shown it can induce silencing and recruit heterochromatic features. We now assess a series of deletions across the transgene for the ability to induce silencing and integrate these results with time-course and chromatin-remodelling inhibitor treatments to follow the steps of XIST-induced silencing and heterochromatinization.

**Discussion:**

We find that in addition to the previously reported necessity of the 5’ A repeat region for XIST-induced silencing, the 1 kb around the small F repeat region and a non-repetitive region at the 3’ end of the RNA are also required to silence genes. Silencing of genes up to 17 Mb from the XIST integration occurs within 2 days, while formation of a Cot-1 depleted domain is slower, and more dependent on the region encompassing Repeat F. The role of this region encompassing Repeat F in both the silencing of actively transcribed genes, the spread of H3K27me3 and the formation of a transcriptionally inert domain suggests a role in a pathway crucial for the spread of XIST across the chromatin to target distal regions of inactivation. Histone deacetylation requires only the A repeat region, with HDAC3 inhibition showing limited effect on silencing, but an impact on H3K27me3 recruitment, and as a result the recruitment of MacroH2A. Global HDAC inhibition impacted silencing in both a distance and dose-dependent fashion. The E repeat region was required for CIZ1 and H4K20me1 recruitment as well as H3K27me3; however, these appeared to act relatively independently. The H3K27me3 mark established by PRC2 integrated silencing and many of the heterochromatic features, while the PRC1 mark ubH2A appeared to be downstream of silencing in these human somatic cells.

**Supplementary Information:**

The online version contains supplementary material available at 10.1186/s13072-022-00438-7.

## Background

The long non-coding RNA (lncRNA) gene XIST was one of the earliest lncRNA genes to be identified and is crucial for the inactivation one of the two X chromosomes in eutherian females to ensure dosage compensation of the genes along the length of the chromosome ([1, 2]; reviewed in [3]). Remarkably, XIST acts as a powerful regulator of chromatin architecture and gene expression solely in *cis*, limiting activity exclusively to the chromosome of origin. Much of the current understanding of XIST activity comes from investigations performed using the mouse as a model system, which allows examination of the process in early development. Thus, we have comparatively less understanding of how the human ~ 19 kb XIST RNA functions than how the ~ 17 kb mouse Xist RNA functions [1]. This research presents novel findings and insights into how human XIST functions, providing new models of how XIST facilitates changes on its chromosome of origin.

The size of XIST contributes to the complexity and reach of its function by allowing integration of transcriptional repressive and chromatin-remodelling processes [4]. While the large size and genomic arrangement of XIST has been retained across species, there is evidence for sequence adaptation over time, with mouse Xist and human XIST sharing 67% sequence conservation. The XIST RNA contains multiple internal tandem repeats labelled A-F which over evolutionary time have either expanded or contracted, and their continued presence across species led to the speculation that they would represent key functional domains for XIST. Of these repeat domains the most studied across species is Repeat A, a highly conserved 429 nucleotide sequence at the 5’ end of the XIST transcript that is thought to form a complex structure [5, 6]. Repeat A is essential for XIST-mediated gene silencing, with previous studies from our group demonstrating that expression of Repeat A alone could silence proximal reporter genes [7]. Studies of mouse Xist have found that the ability of Repeat A to induce gene silencing depends upon its ability to activate histone deacetylases (HDACs) and in particular it has been shown that the activity of HDAC3 to remove the acetylation groups from histone tails and thereby remove signals for active gene expression is critical for Xist-mediated gene silencing. The Repeat A region has been shown to bind multiple proteins [8, 9]. Notably the binding of SPEN was reported in mouse to activate HDAC3 through recruitment of NCOR [10]. The related RBM15 that also binds the A repeats, also recruits deposition of m6A on XIST which has also been reported to be critical for silencing [11], and may be another outcome of SPEN binding [12].

In addition to the loss of histone marks and chromatin features associated with active transcription, XIST is well-known to enrich its chromatin with a broad array of heterochromatin-associated marks and factors. We previously reported that for human XIST the enrichment of H3K27me3 by PRC2 was crucial for the subsequent enrichment of MacroH2A at the site of the XIST RNA cloud within the nucleus. Similarly, enrichment of the heterochromatin mark H2AK119ub (ubH2A) by PRC1 was essential for the enrichment of SMCHD1 [13]. Surprisingly, multiple distinct domains were required for the enrichment of all four of these heterochromatin marks, and in human somatic cells the mechanisms governing PRC1 and PRC2 recruitment/activation by XIST are different from the pathway established in mice where PRC1 recruitment through hnRNPK interactions with the region of Xist surrounding Repeats B and C is essential for the recruitment of PRC2. In addition to the extensively studied roles of PRC1 and PRC2, an ongoing question has been what role the well documented but poorly understood XIST-associated heterochromatin feature H4K20me1 plays in the overall process of XIST-mediated chromosome inactivation. The monomethylation of H4K20 is established by KMT5A (also known as PR-SET7, SETD8 & SET8) and was one of the very first chromatin features to be observed on the inactive X chromosome. Recently it has been shown that H4K20me1 accumulates rapidly during XCI in mouse models, and that in mice there is clear evidence that its accumulation also depends upon Xist repeats B and C [14]. It is therefore of great interest whether the spread of H4K20me1 in human cells also depends upon these domains, or whether the human and mouse pathways have also diverged.

CIZ1 is one of a group of matrix-associated factors shown to interact with XIST and has been observed to become strongly enriched at the site of XIST RNA when examined in both human and mouse models [15]. It has been proposed that CIZ1 binds directly to XIST and acts to help maintain the localization of XIST during XCI. In mice it has been shown that CIZ1 interaction with Xist RNA depends specifically upon Repeat E, one of the regions found to be essential for H3K27me3 in our previous work. It was therefore of great interest to us to identify which regions of human XIST are essential for interactions with CIZ1 and whether this recruitment depended upon the activity of previously established XIST-associated chromatin-modifying factors.

In order to examine if and how these various XIST-interacting factors and pathways are associated with each other, we used the previously established HT1080-inducible XIST model system described in previous studies [13, 16, 17]. A full-length XIST cDNA transgene in these male cells is integrated as a single copy on an autosome (8p) and induced with doxycycline (dox). Advantages of this model system include that effects of de novo XIST expression are studied in a male cell line, where the inducible XIST transgene is the only source of XIST RNA, without the interference of XCI-associated factors that have been shown to influence or adapt the activity of XIST at its endogenous location. Our previous study found that the chromatin-remodelling pathways initiated by XIST were conserved when XIST was expressed from 8p or an integration site on the X chromosome [13]. Due to the differences in the context where XIST is being induced in the HT1080 adult male fibrosarcoma cell line, we do not observe the same level of stable silencing observed during endogenous XCI, as once XIST stops being induced with doxycycline we observe a gradual reactivation of previously silenced reporter genes [17]. Previous work has established that even when expressed from 8p, XIST is capable of forming a clear RNA ‘cloud’ within the nucleus, causing allele-specific gene inhibition and forming a transcriptionally inert domain within the nucleus. This transcriptionally inert domain can be visualized by fluorescent labelling of human Cot-1 through fluorescent in situ hybridization (FISH) [18], and thus is referred to as a Cot-1 hole. Furthermore, inducing XIST expression from 8p leads to the accumulation of many of the heterochromatin marks associated with XIST activity in its endogenous context, including the enrichment of H3K27me3, ubH2A, MacroH2A, SMCHD1 and H4K20me1. In our previous publication, we described a series of partially deleted XIST transgenes that we had created using CRISPR/Cas9 [13]. These deletions were designed to allow for every region of XIST to be specifically tested for the effect it had on the pathways initiated by XIST and to allow us to create a map of the function of each part of the XIST transcript. The deletions range from 630 to over 3 kb in size and are generally named according to repeat regions contained therein. However, more than the repeat is removed, of note repeat F is only two 42-bp repeats within an approximately 1-kb deletion, Bh is less than 100 bp of an over 800-bp deletion, the D repeat contains degenerate repeats beyond those deleted, and repeat E is only approximately one-third of the Repeat E deletion. The sequencing data and validation of those deletions were published in that previous article, as well as the relative expression levels of XIST RNA transcripts produced by each deleted cell line [13]. Induction of XIST in most of the transgene deletion cell lines with doxycycline for 5 days produced levels of XIST RNA comparable to their Full XIST progenitor. The Delta 3’ deletion construct produced statistically less XIST RNA when induced, though the deletions still demonstrated an ability to recruit H3K27me3 and MacroH2A that was comparable to Full XIST.

In this study, we explore the ability of the XIST transgene deletions to induce gene silencing through examining both allele-specific silencing and Cot-1 hole formation, as well as the additional features of histone deacetylation, H4K20me1, and CIZ1 enrichment. We observe that the changes to chromatin architecture lag behind the allele-specific silencing induced by XIST. Overlap of essential domains was observed, and to assess the directionality of potential interactions we used small molecule inhibitors affecting the enzymatic activity of the chromatin-remodelling complexes PRC2, PRT1, KMT5A and HDACs. This work demonstrates a clear but complex connection between chromatin remodelling and gene silencing and the results presented here suggest that distinct regions of XIST are crucial for multiple different steps in the process of chromatin remodelling.

## Methods

### Cell lines and culturing

The HT1080 (male fibrosarcoma) cell lines with doxycycline-inducible full-length XIST cDNA constructs integrated into chromosome 8p have been described in previous publications [16, 17]. The XIST CRISPR-mediated deletion transgenes studied throughout this work were all created by targeted excision of specific sequences from the full-length inducible XIST cDNA construct as described previously [13]. The additional deletion constructs Delta PflMI, Exon 1, and Delta Delta, were generated through independent integration of partial XIST sequences into an 8p FRT integration site in the HT1080 cell line [17]. All XIST inducible constructs were under the control of a CMV promoter with a Tet repressor element as described previously [13, 17]. Induction of XIST was performed with 1 µg/ml doxycycline, refreshed daily. Cells were cultured at 37 °C in 5% CO_2_ in Dulbecco's modified Eagle's medium (DMEM) supplemented with 10% fetal calf serum (Sigma-Aldrich) by volume, 100 U/ml penicillin–streptomycin, non-essential amino acids and 2 mM l-Glutamine.

All of the chemical inhibitors used in this assay were first dissolved in DMSO and then added to culture media at the desired concentration. Inhibition of HDAC3 was performed using the chemical RGFP966 (Sigma-Aldrich) and the inhibition of all HDACs non-specifically was performed using trichostatin A (TSA; Sigma-Aldrich). Inhibition of the catalytic activity of KMT5A (also known as PR-SET7/SET8/SETD8) was performed using the chemical ryuvidine from Abcam. Inhibition of PRC2 and PRC1 was performed using GSK343 and PRT4165 (both from Sigma-Aldrich) as described previously [13].

### RNA isolation and reverse transcription

The isolation of RNA, its purification and reverse transcription were all performed using the same methodology described in our previous publication in order to ensure consistency of results [13]. In brief, RNA was extracted and purified from cells with TRIZOL according to the protocol of the manufacturer, Invitrogen. 5 µg of RNA was treated with DNAse1 from Roche according to the manufacturer’s protocol: 5 µl 10 × DNAse1 buffer, 1 U/µl RNAseI, 10 units of DNAse1 then the volume was raised to 50 µl with DEPC-treated ddH_2_O. The DNASe1-treated RNA was then reverse transcribed using M-MLV Reverse Transcriptase from Invitrogen. The reaction volume consisted of 1  µg of RNA, 4 µl of 5 × first strand buffer, 0.25 mM dNTP, 0.01 mM DTT, 1  µl random hexamers, 1U/ul RNAse Inhibitor and 1 µl of M-MLV with DEPC ddH_2_O making up the remainder of the 20 µl volume. Reverse transcription was incubated for 2 h at 42 °C then heat inactivated.

### Pyrosequencing

Prior to pyrosequencing, the cDNA of the four genes of interest were amplified by PCR. The amplification of these cDNA sequences was performed using Taq DNA polymerase from Invitrogen. PCR mixtures were prepared with 0.2 µl 25 mM dNTPs, 0.75 µl 50 mM MgCl_2_, 2.5 µl 10 × Buffer, 25 nmol sense and antisense primers, one of which was biotinylated, 1 µl of cDNA template, 0.125  µl Taq DNA Polymerase and then brought up to a final volume of 25 µl with ddH_2_O. All the PCRs of pyrosequencing primer pairs were performed at a melting temperature of 58.3 °C and extension times of 1 min. Two technical replicates consisting of 12 µl of PCR product were used in each pyrosequencing reaction in a PyroMark MD machine from Qiagen using CDT tips according to the recommended protocol. The PCR products were combined with 38 µl of PyroMark’s binding buffer, 35 µl of ddH_2_O and 2 µl of Streptavidin Sepharose High Performance beads from GE Healthcare. The mixture was then agitated at 1400 rpm to allow the DNA to bind to the beads. While the PCR/bead mixture was shaking, 0.144 µl of 25 nmol/µl SNP primer was combined with 11.856 µl of PyroMark Annealing buffer in the well of a PyroMark optical plate. The containers of the PyroMark workstation were filled with the following: 1 with 100 ml of 70% ethanol, 2 with ~ 100 ml denaturing solution, 3 with ~ 120 ml of wash buffer and 5 with ~ 100 ml of ddH_2_O. The workstation comb was rinsed in ddH_2_O to remove any residual dust or contaminants, then the PCR product containing mixture was sucked up through the comb of the vacuum apparatus. The DNA and beads remained held against the outside of the semipermeable membrane of the comb by the negative pressure generated by the vacuum apparatus. As soon as the liquid was sucked up, the comb was gently placed in each of the numbered 1–3 containers of the workstation, allowing the PCR product coated beads to be treated with the various liquids. The comb of the vacuum apparatus was then carefully lowered directly over the optical plate, without touching the primer mixture. The vacuum was then disconnected and the teeth of the comb were lowered directly into the wells of the optical plate, swirling gently to ensure that the beads were released into the primer-containing annealing mixture. The combs were then carefully removed, and the optical plate was heated to 80 °C for 2 min. The plate was then loaded into the PyroMark MD machine and the CDT tips were filled with their respective primers, enzyme and substrate components from Qiagen. The PyroMark software was used to run the PyroMark MD machine and calculate the allelic ratio present within the PCR product. The relative strength of XIST-induced silencing was measured by comparing the change in allelic contribution in a condition of interest to the change in allelic contribution caused under control conditions. The control conditions were generally Full XIST induced for the same period of time as the condition being tested (either 2 days or 5 days). By expressing the strength of silencing as a normalized value to a control population, it provided us with a convenient way to express how effectively XIST silenced distal genes. A value of 1 indicated that the change induced by XIST under testing conditions was comparable to control full-length XIST and 0 indicated that XIST was unable to induce silencing in those conditions. Differences between groups were tested using the *t*-test with multiple testing correction of thresholds of significance. The primers used for this protocol are shown in Additional file 1: Table S8.

### qPCR

Quantifying the expression of XIST across the cell lines described here was performed using quantitative real-time PCR (qPCR) with the Maxima Hot Start Taq from Thermo Scientific and EVAgreen from Biotium. The amplification reaction was performed on StepOnePlus Real-Time PCR machines from Applied Biosystems and analysed on the accompanying software. Three technical replicates were performed for each biological replicate studied. Diluted cDNA for each biological replicate was amplified in 20 µl reaction volumes consisting of 0.2 mM dNTP, 5 mM MgCl2, 0.25 nmol of sense and antisense primers, 2 µl of 10 × Maxima Hot Start Taq Buffer, 0.16 µl of Maxima Hot Start Taq, 1 µl of EVAgreen and 17 µl of ddH_2_O. The reactions were performed in MicroAmp Fast Optical 96-Well Reaction Plates from Applied Biosystems. The PCR machines initially heated the plate to 95 °C for 5 min before running through 40 cycles of 95 °C for 15 s, 60 °C for 30 s and finally 72 °C for one minute. Relative levels of XIST were compared to two endogenous control genes, *PGK1* and *UBC*, and control 5ddox Full XIST cell lines were always run as controls on each plate. The relative quantification of XIST was performed by calculating the ΔΔCT = (CT_XIST_test_−CT_endogenous_test_)/(CT_XIST_control_−CT_endogenous_control_) and expressed as rq = 2^−ΔΔCT^. The primers used for this protocol are shown in Additional file 1: Table S8.

### IF-FISH and FISH

The detailed protocol for all of the IF-FISH protocols described in this paper can be found in our previous publication [13] as well as online at protocols.io: dx.doi.org/10.17504/protocols.io.bqtdmwi6. The protocol is summarized below. The antibodies used for this protocol are listed in Additional file 1: Table S9.

Adherent cells were permeabilized with Triton-X and fixed with paraformaldehyde. Cells were incubated with primary antibody solutions consisting of 1 µl of antibody in 100 µl of RNAse inhibited PBT for 4–6 h. After the primary antibody incubation the coverslips were washed to remove unbound antibodies. The coverslips were then incubated for an hour in a secondary fluorescently labelled antibody solution (1 µl of secondary antibody solution in 100 µl of RNAse inhibited PBT) for 1 h. After this incubation, the coverslips were washed again and fixed for 10 min with paraformaldehyde. The coverslips were then incubated at 37 ℃ overnight with fluorescent probes targeting XIST RNA. The FISH solution consisted of 10 µl of Cot-1 DNA, 2 ul of salmon sperm DNA and 5 µl of fluorescent probes created by nick translation according to Abbott Molecular’s protocol. The mixture of probes and decoy DNA was suspended in 20 µl of equal parts hybridization solution (20% BSA and 20% dextran sulfate in 4 × SSC) and deionized formamide from Sigma. The next day coverslips were incubated for 20 min in a mixture of deionized formamide and SSC (Invitrogen). The coverslips were washed in 2 × then 1 × concentrations of SSC for 20 min each then incubated for 15 min with a DAPI methanol solution. Microscopy was performed using laser excitation of fluorophores with a confocal microscope using a 100 × objective lens.

Fluorescent intensity of the different colour channels was measured using ImageJ (Fiji) and the BAR plugin. Bisections of the nucleus that intersected the point of maximum XIST fluorescent intensity were used to calculate the relative enrichment or depletion of a given heterochromatin mark/protein at the site of XIST. The standard score (*z*-score) for each cell was calculated by comparing the average fluorescent intensity of the secondary antibody fluorophore at the regions where XIST fluorescence was greater than 50% of the maximum to the average level at sites where XIST fluorescence was < 25%. The average levels of the secondary antibody fluorescence and standard deviation were calculated using the program R, which were used to determine the *z*-score for each individual cell. Sixty cells were analysed for every condition, and the distribution of the 60 *z*-scores for each set of conditions was compared using Mann–Whitney (M.W.) U test with multiple testing corrections.

FISH that was performed with Cot-1 fluorescently labelled with dUTPs coupled to a red Alexa Fluor 598 and XIST RNA probes containing green dUTPs coupled to Alexa Fluor 496 from Enzo. Similar to what was described above a FISH solution was created by combining 10 µl of red Cot-1 probe and 5 µl of green XIST probe along with 2 µl of Salmon sperm DNA. This combination of DNA and probes was dissolved in 10 µl of formamide and 10 µl of hybridization buffer. The coverslips were incubated in the FISH solution overnight at 37 ℃ in a humid chamber to prevent evaporation. The FISH protocol was identical to the IF-FISH protocol detailed above from that point onwards.

### Western blotting

The protocol used for protein extraction and western blotting is shown in detail in our previous publication [13] and online at protocols.io: dx.doi.org/10.17504/protocols.io.bqubmwsn. The antibodies used for this protocol are listed in Additional file 1: Table S9.

In brief, protein was extracted from cells using RIPA buffer according to the Cold Spring Harbor protocol. Protein was treated with SDS-PAGE and run on a 15% acrylamide gel. Protein was then transferred to 0.2um nitrocellulose paper from ThermoFisher. After transfer, the nitrocellulose membrane was treated with a blocking buffer (0.1% v/v Tween-20 and 3% bovine serum albumin) and then after washing with TBST was incubated overnight in a primary antibody solution overnight at 4℃. The next day, the nitrocellulose was washed to remove unbound primary antibodies with TBST and incubated for 1 h in a secondary antibody solution. The nitrocellulose membrane was then imaged at the relevant wavelengths using a LI-COR Odyssey machine and Image Studio software package from BioAgilytix.

## Results

### Transcriptional repression requires both repeats A and F as well as 3’ non-repeat region

**Fig. 1:**
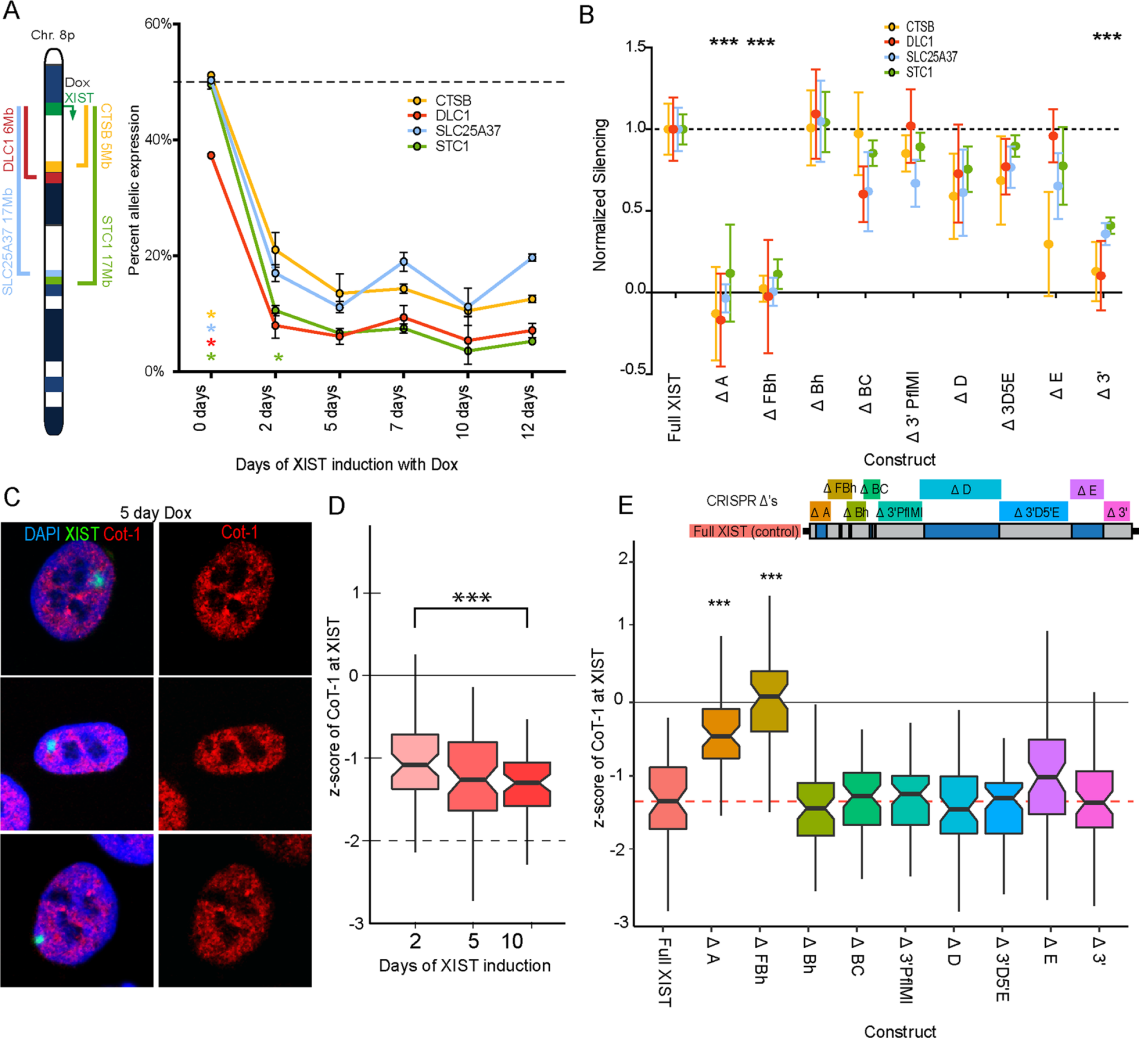
XIST-mediated silencing depends on Repeats A and F as well as 3’ non-repeat domain. **A** The change in allelic contribution over time of four 8p genes repressed by the induction of the XIST cDNA construct in the HT1080 cell line are shown. Three biological replicates were tested for each time point and significance was calculated using a student *t*-test relative to 5ddox. **B** The change in allelic contribution (for the same genes as in A) caused by 5 days of induction of XIST deletion constructs are shown normalized to the full-length XIST construct. A value of 1 indicates that the strength of silencing was comparable to Full XIST, a value of 0 indicates no silencing occurred. Deletion constructs where all 4 alleles statistically differed from Full XIST by *t*-test with multiple testing correction are indicated. **C** FISH images of XIST (green) and Cot-1 (red) and DAPI (blue) used to exemplify the formation of a transcriptionally inert “Cot-1 hole” in Full XIST HT1080 cells treated with dox for 5 days. **D** The depletion of Cot-1 RNA at the site of XIST RNA was calculated as the standard score (*z*-score) for each of the 60 cells examined at 2, 5 and 10 days of induction. Cell populations were blinded through the process and the distribution of conditions were statistically compared using a Mann–Whitney test with multiple testing correction. There was no significant change across the timepoints. **E** Each deletion construct induced for 5 days was tested for its relative depletion (*z*-score) of Cot-1 RNA hybridization at the site of XIST. Samples were blinded and statistically compared to Full XIST using a Mann–Whitney test with multiple testing correction. **A**–**E** The thresholds for statistical significance of all p values are: * < 0.05, ** < 0.01 and *** < 0.001, with these thresholds adjusted based on the number of tests

The mechanisms by which XIST functions as a *cis*-acting repressor of gene expression are not yet understood, particularly in humans. To follow the dynamics of XIST-mediated transcriptional repression, we examined allelic silencing upon XIST induction. While it has been shown that induction of the region of XIST encompassing Repeat A is sufficient to silence an adjacent reporter gene, it is not known what elements are necessary for XIST to silence distal genes [7, 17]. Four genes with coding SNPs megabases from the site of the XIST transgene integration into chromosome 8p were identified: *CTSB* (~ 5 Mb), *DLC1* (~ 6 Mb), *SLC25A37* (~ 17 Mb) and *STC1* (~ 17 Mb). While silencing of 8p genes further away has been observed by RNA-seq analysis, these four genes were studied as they have coding SNPs and have been well-validated to consistently become silenced following XIST induction in this model [16]. Allele-specific silencing was measured by pyrosequencing of a region of the cDNA of these genes containing a SNP before and after the induction of the XIST cDNA transgene with dox. Without XIST induction the two alleles contributed approximately equally, and the strength of silencing of a specific allele was determined by its decreased representation following XIST induction. The change in allelic contribution was measured at six selected intervals between 2 and 12 days of XIST induction (Fig. 1A and Additional file 1: Table S1). Within 2 days of XIST cDNA induction, we observed a substantial reduction in allele-specific contribution across these four genes. Three of the four genes were silenced at day two to levels statistically comparable with the maximal levels observed (Fig. 1A). Only the most distal gene, *STC1*, differed significantly at day two of XIST induction from the subsequent levels at later time points, indicating that silencing was still underway. By the day five time point, maximal levels of allelic silencing were evident at all alleles (Fig. 1A).

We then assessed the impact of nine different CRISPR-mediated deletions spanning the full XIST transgene (Fig. 1B and Additional file 1: Table S2 (see ref. [13]). The change in allelic ratio observed for each transgene deletion cell line was normalized to the change in allelic ratio for full-length XIST and presented as strength of silencing, where a value of 1 indicated comparable silencing to Full XIST and 0 indicated no change in allelic contribution from the pre-induction levels. The ~ 1.7-kb region spanning both Repeats A and F was found to be critical for silencing of all four alleles examined as there was no change in relative allelic contribution following induction of either the Delta A or Delta F constructs (Fig. 1C, Additional file 1: Table S3). Additionally, a ~ 630-bp region at the 3’ end (Delta 3’) also significantly impacted allele-specific silencing. Deletion constructs previously generated by FLP-mediated re-integration were consistent with the CRISPR deletion constructs with loss of the large 3.8 kb internal region of exon 1 not affecting silencing but the loss of the region corresponding to the post exon 1 sequence (3.6 kb) replicating the deficits in silencing observed in the Delta 3’ construct (Additional file 2: Fig. S1).

The ability of XIST to form a Cot-1 hole (a transcriptionally inert three-dimensional domain within the nucleus) was examined by RNA FISH using Cot-1 as a probe (Fig. 1C) which hybridizes to the heteronuclear transcripts of the genome [19]. Quantification was performed by line measurement of fluorescence intensity across nuclei and calculating the standard score (z-score) of Cot-1 fluorescent intensity at the XIST RNA cloud, as previously described [13]. The transcriptionally inert domain generated by XIST had not reached its maximal level by day 2 (median z-score = 1.06, Fig. 1D). This suggested that the nuclear reorganization mediated by XIST to create a transcriptionally inert domain lagged slightly behind the allele-specific silencing observed in Fig. 1A. Across the various transgene deletions, again the largest impact was from regions encompassing Repeats A and F (*p* < 1.0 × 10^–13^, Fig. 1F and Additional file 3: Fig. S2). Surprisingly, we observed that the ~ 1-kb region encompassing Repeat F (*z*-score = 0.182) to be slightly more important than Repeat A (*z*-score − 0.483) for forming the transcriptionally inert domain (*p* = 2.1 × 10^–05^). No other region of XIST was found to be critical for the formation of this transcriptionally inert nuclear domain suggesting that the 5’ region of XIST is specifically essential for this process.

The two methods of measuring transcriptional repression confirmed the critical role for the A repeat region in silencing, and also demonstrated a similarly critical role for the nearby F repeat region. However, there were also surprising differences between the approaches. The dispensability of the 3’ most region of XIST for the formation of a transcriptionally inert domain suggested that additional factors at the 3’ end of XIST are contributing specifically to the silencing of actively expressed genes. Genic silencing was accomplished quickly after XIST induction, while Cot-1 suppression accumulated over time. We have previously seen that recruitment of PRC1/2 and their affiliated marks is maximal at day 5 or later [13], so the recruitment of other heterochromatic marks and the loss of euchromatin may contribute to Cot-1 suppression.

### Acetylation most resembles silencing in requirement for critical XIST domains

**Fig. 2:**
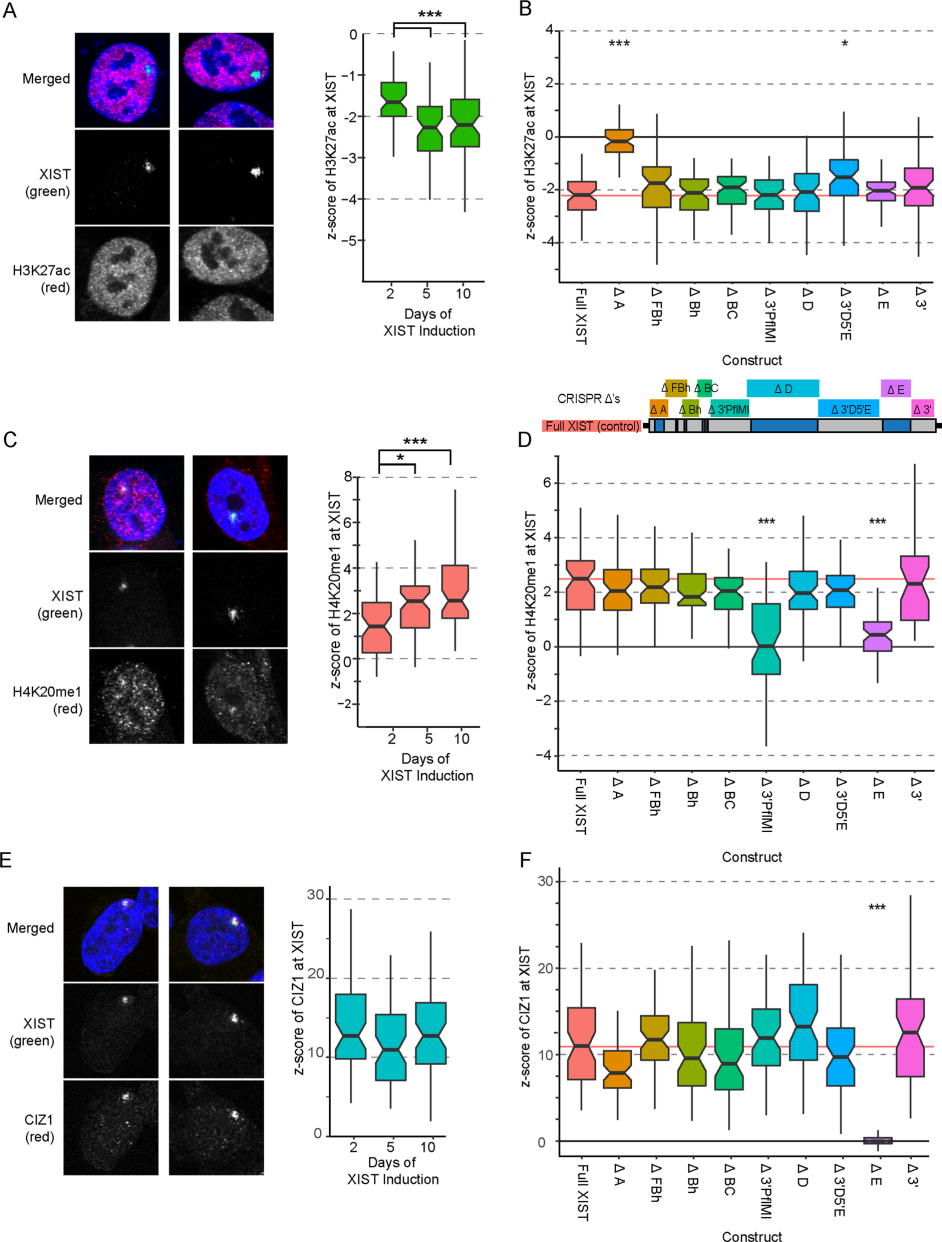
The regions of XIST critical for histone deacetylation and enrichment of H4K20me1 and CIZ1. **A** Example images showing typical depletion of H3K27ac (red) at the site of XIST RNA after five days of induction in the HT1080 cell line and *z*-score s showing the depletion of H3K27ac over time by XIST induction. **B** Each deletion construct induced for 5 days was tested for its relative depletion (*z*-score) of H3K27ac at the site of XIST (59–60 cells per construct). The distributions of *z*-scores for each construct are shown in boxplots with significance calculated relative to the Full XIST control. **C** Similar to A, example images and time point analysis were performed for the enrichment of H4K20me1 at the site of Full XIST expression in the HT1080 cell line. **D** Similar to B, the deletion constructs were tested for their enrichment of H4K20me1 after 5 days of XIST induction and the population distributions of the 60 cells analysed are shown in boxplots. **E** Example images of the enrichment of CIZ1 at the site of XIST and the population distribution of the enrichment profile of cells (*z*-score) at various induction timepoints. **F** The plotted enrichment profiles of CIZ1 at XIST in each of the deletion constructs. **A**–**F** All samples were blinded throughout the analysis process and statistical significance calculations were performed using the Mann–Whitney test with the thresholds for statistical significance of all p values being: * < 0.05, ** < 0.01 and *** < 0.001, with these thresholds adjusted based on the number of tests

Since silencing occurred before maximal recruitment of the heterochromatic marks (H3K27me3 and ubH2A) previously examined [13], we explored additional chromatin marks or proteins described to be established by the XIST RNA. As previous studies have linked the loss of histone acetylation with the loss of transcriptional activity, we set out to examine the dynamics of histone acetylation loss at the nuclear domain of XIST and determine whether we could observe a depletion of histone acetylation using this model system and to identify the regions of XIST critical for this deacetylation. Depletion was detectable by day 2 (*z*-score = − 1.62), but was greater by day 5 (*z*-score = − 2.16) by which point it plateaued and did not accumulate further by day 10 (*z*-score = − 2.17, Fig. 2A and Additional file 1: Table S1). Analysis of the transgene deletions for their depletion of histone acetylation (H3K27ac) at the XIST focal accumulation was performed using the same method used to examine the depletion of Cot-1 and it was observed that only loss of the A repeat in the Delta A deletion disrupted XIST-mediated histone deacetylation (*z*-score = − 0.17, *p* = 2.6 × 10^–18^, Fig. 2B, Additional file 4: Fig. S3 and Additional file 1: Table S4). Previous studies examining the regions of XIST critical for the recruitment of the PRCs [13] had observed numerous interdependent domains across the length of XIST, suggesting that HDAC recruitment and/or activation by XIST operated at least semi-independently of the PRCs.

In addition to the more extensively studied pathway of XIST-mediated histone deacetylation, we wished to examine the enigmatic role of H4K20me1, established by KMT5A. While one of the earliest marks observed to be enriched on the inactive X chromosome [20], it has been studied less than the other marks described so far and little is known of the pathway regulating its activity. Analysis of H4K20me1 across three time points revealed that the mark plateaued statistically at day five as reported for the PRC mediated marks described previously (5- and 10-day *z*-score *s* =  ~ 2.5, Fig. 2C and Additional file 5: Fig. S4 and Additional file 1: Table S1). The regions of XIST crucial for H4K20me1 enrichment were examined using the XIST transgene deletion cell lines. The enrichment profiles for the 60 cells examined in each case were compared to the Full XIST control population (Fig. 2D and Additional file 1: Table S4). We observed that loss of the non-repeat region between the C and D repeat region disrupted the enrichment of H4K20me1 by XIST, as did the loss of the region including Repeat E. Loss of any other region of XIST did not impair enrichment of H4K20me1 (mean *z*-score s ≥ 2, Fig. 2D).

Repeat E has been reported to be important for XIST localization through recruitment of CIZ1 and associated condensate-forming RNA binding proteins [21]. We thus extended our analysis to include CIZ1 and observed a strong co-localization with XIST that remained constant across all time points (Fig. 2E and Additional file 1: Table S1). Consistent with other studies, CIZ1 recruitment was solely dependent on the presence of the E repeat region (*z*-score = − 0.03, *p* = 3.6 × 10^–21^ Fig. 2F, Additional file 6: Fig. S5 and Table S4). CIZ1 requires only this single domain of XIST to be recruited and was fully enriched by the earliest time point (2-day *z*-score = 12.9, Fig. 2E and F, Additional file 1: Table S1 and S4).

Overall, examination of the domains of XIST required to recruit features showed that silencing and loss of acetylation both required repeat A; while H4K20me1 and CIZ1 recruitment to the XIST domain had no overlap with domains required for silencing, although they both required the E repeat region. Enrichment of the majority of proteins and histone marks to the XIST domain did not appear to be critical for silencing. In addition to the well-studied Repeat A region, we also observed that the ~ 1-kb region encompassing Repeat F and a 3′ 630-bp region were critical for silencing. While those domains were not required for H4K20me1 or CIZ1 recruitment, we previously demonstrated that H3K27me3 enrichment required the Repeat F surrounding region, while the 3’ region was critical for ubH2A recruitment [13].

### Chromatin remodelling by PRC2 and KMT5A impacts XIST-mediated silencing

As previous studies have suggested linkages between the ability of XIST to induce gene silencing and chromatin-remodelling pathways, we set out to examine what connections exist within this system in which XIST is induced in differentiated cells. The domains of XIST required for silencing overlap domains required for the chromatin features established by XIST, however it remains to be determined whether these pathways are interdependent. To test the role of the chromatin-modifying complexes previously associated with XIST-mediated activity, we used small molecule inhibitors that impacted the enzymatic activities of these complexes without disrupting the levels of the component proteins.

**Fig. 3:**
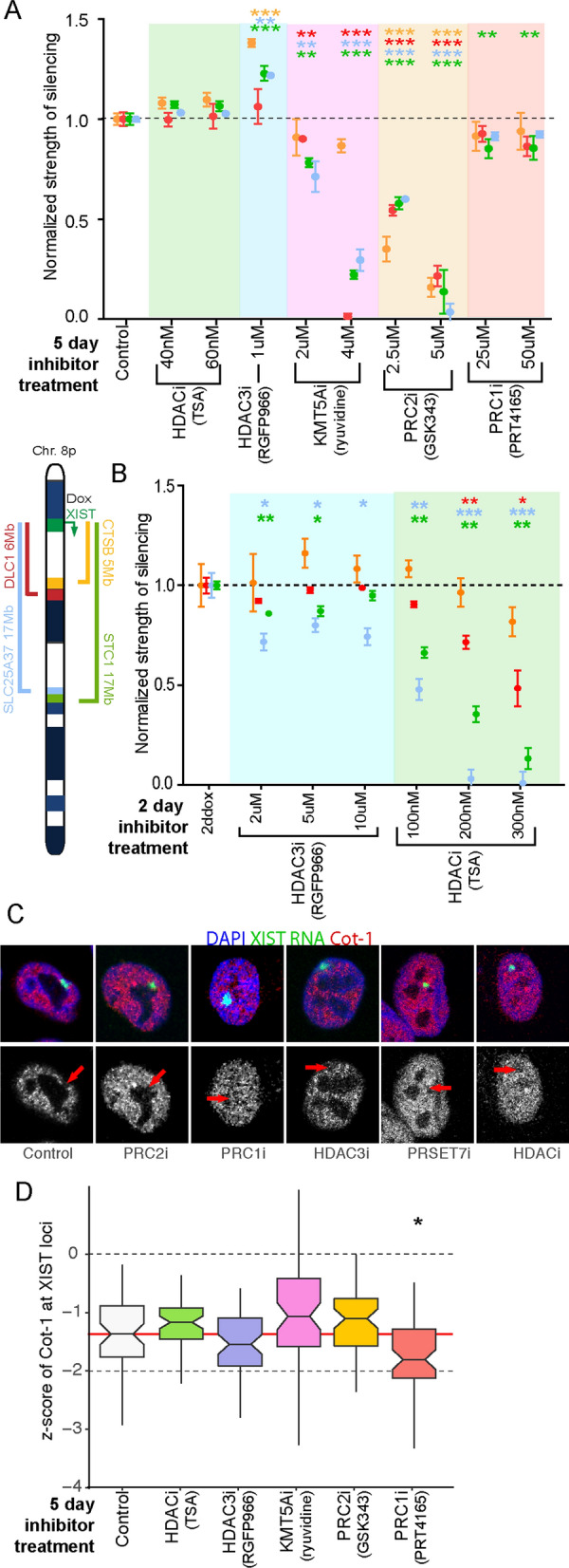
The role of chromatin modification on XIST-induced gene silencing. **A** The ability of Full XIST to induce allele-specific repression at four distal genes is shown when cells were treated with chemical inhibitors for XIST-associated chromatin remodelers. Three biological replicates were tested for each condition shown and statistical significance of a treatment’s effect on XIST-induced silencing was calculated using a *t*-test with multiple testing correction. The concentration of chemical inhibitors in media along with the factor inhibited are listed, and the key for the genes assessed is beside panel **B**. **B** Test of whether HDACs contribute to silencing if used at much higher concentrations for only 2 days, as longer exposure was lethal to cells. Strength of silencing for these higher HDACi treatments was normalized and compared to uninhibited 2ddox Full XIST by *t*-test. **C** Example images depicting XIST (green) mediated formation of a transcriptionally inert domain (Cot-1, red) over the course of five days of induction combined with chemical inhibition. **D** Boxplot showing distribution of Cot-1 depletion (*z*-score s) across populations of 60 cells, following 5 days of chemical inhibition and Full XIST induction. All conditions were blinded through the process and statistical comparisons of population distributions of *z*-score s performed using the Mann–Whitney test. **A**–**D** Chemical inhibition was performed using the following: TSA (HDACi), RGPF966 (HDAC3i), ryuvidine (KMT5A), GSK343 (PRC2i) and PRT4165 (PRC1i). The thresholds for statistical significance were all corrected for multiple testing correction from * < 0.05, ** < 0.01, *** < 0.001 (p values listed individually in Additional file 1: Table S5)

Cells were treated for 5 days in dox in combination with inhibitors. We anticipated that silencing might be sensitive to histone deacetylation given that acetylation required the A repeat region, which is essential for silencing in both human and mouse. Inhibitors were initially tested across multiple concentrations to determine the highest functional concentration in which the cells could survive 5 days. The H3K27ac antibodies used for IF proved unsuitable for examining levels of histone acetylation by western blotting, so we instead used H4K8ac antibodies validated and gifted to us by the Howe lab at UBC. For the broad-spectrum HDAC inhibitor TSA the highest concentration (60 nM) resulted in over a fourfold increase in histone acetylation (H4K8ac) when treated cells were compared to control populations through western blotting (Additional file 7: Fig. S6A). A dose-dependent effect of TSA was observed across the lower doses, with the 40 nM TSA treatment showing a greater than twofold relative increase in histone acetylation compared to controls (Additional file 7: Fig. S6A). Despite the increase in histone acetylation detected by western, analysis of allelic silencing mediated by 5 days of full-length XIST expression from 8p demonstrated no perturbation in any of the genes examined and did not significantly disrupt XIST expression (Fig. 3A and Additional file 8: Fig. S7 and Additional file 1: Table S5). In mouse development it has been suggested that XIST-mediated silencing via the A domain depends upon HDAC3, and thus we used RGFP966, previously shown to specifically inhibit HDAC3. Again, no reduction in silencing was observed at concentrations in which cells were viable for 5 days though a subtle but statistically significant increase in the strength of silencing of CTSB, SLC25A37 and STC1 was observed (*p* < 4.9 × 10^–5^, Fig. 3A and Additional file 1: Table S5). Treating cells with HDAC inhibitors in the absence of dox did not affect the allelic ratios (Additional file 9: Fig. S8).

To explore whether other chromatin modifications had any impact on silencing, we also used ryuvidine, a known KMT5A inhibitor [22], which at 2 uM reduced H4K20me1 levels dramatically to 25% of control levels while the highest dose used, 4uM, reduced levels to 3% of control levels (Additional file 7: Fig. S6B). Both doses resulted in a marked increase in XIST levels, though these failed to reach statistical significance (Additional file 8: Fig. S7). At 2 uM ryuvidine there was limited impact on silencing for three of the four genes examined (*p* < 2.2 × 10^–4^); with the closest gene (*CTSB*) still able to silence (strength of silencing = 86%, *p* > 0.33, Fig. 3A, Additional file 1: Table S5). At the higher dosage of 4 uM ryuvidine the loss of silencing was significant for all four genes, although still less for *CTSB* (*p* = 8.3 × 10^–3^; Additional file 1: Table S5).

We also inhibited both the polycomb complexes as previously [13] and now examined the effect on gene silencing. Our previous examination of the PRC2 inhibitor GSK343 had shown impact on XIST levels at the highest doses (Additional file 8: Fig. S7), so here we examined the 2.5uM and 5uM concentrations (Fig. 3A and Additional file 1: Table S5). At 2.5 uM, we observed a significant effect on XIST-mediated gene silencing while halving global H3K27me3 levels and a near total loss of silencing at the higher dose of 5 µM when H3K27me3 levels were ~ 25% of pre-treatment levels (*p* < 6.0 × 10^–4^, Fig. 3A, Additional file 1: Table S5, Additional file 7: Fig. S6C). Inhibition of PRC1 using the chemical inhibitor PRT4165 across 25uM and 50 µM concentrations for 5 days produced a dose-dependent decrease in levels of ubH2A to 55% of control levels at 50 µM (Additional file 7: Fig. S6D). Higher concentrations of PRT4165 resulted in extensive cell death. No substantial effect of this inhibitor at either concentration was observed either on the ability of XIST to induce allelic silencing or in levels of XIST as described previously (also, Additional file 8: Fig. S7A). The weak decrease in strength of silencing of STC1 was significant (*p* < 2.8 × 10^–4^, Additional file 1: Table S5).

Previous studies have associated the ability of XIST to remove the acetylation marks that promote active transcription with the silencing induced by XIST. As we had observed HDAC inhibition at five days of treatment did not weaken XIST-mediated silencing, we examined whether higher doses at the 2-day time point could yield insights into whether XIST-mediated silencing was dependent on deacetylation. None of the treatments resulted in a statistically significant effect on XIST levels (Additional file 8: Fig. S7B). Neither the 5uM or 10uM treatment of the HDAC3-specific inhibitor, RGFP966, impacted allelic silencing statistically once multiple testing correction was introduced (*p*-values > adjusted threshold of 8.3 × 10^–3^), further suggesting that HDAC3 catalytic activity is not essential for silencing in this context (Fig. 3B, Additional file 1: Table S5). Treatment with higher concentrations of the broad-spectrum HDAC inhibitor, TSA, caused a dosage and distance-dependent effect on XIST mediated silencing (Fig. 3B, Additional file 1: Table S5). The two most distal genes, *SLC25A37* and *STC1*, both differed significantly in their allelic distribution at all doses of TSA examined compared to controls (*p* < 8.9 × 10^–6^, Additional file 1: Table S5). At the two highest treatment doses, 200 nM and 300 nM, silencing at the next most distant gene, DLC1, was significantly attenuated (*p* < 3.6 × 10^–4^, Additional file 1: Table S5). Silencing of the closest gene to the XIST construct, *CTSB*, remained statistically comparable across all treatments, indicating that loss of HDAC activity did not impact the ability of XIST to repress this gene.

The ability to form a transcriptionally depleted compartment in the space occupied by XIST RNA (visualized with RNA specific fluorescent probes) was monitored with RNA FISH using Cot-1 RNA as a probe. This formation of the Cot-1 hole was also impacted by loss of the A repeat region of XIST, but more strongly impacted by loss of the region surrounding the small F repeat, suggesting some independence from the genic silencing monitored by pyrosequencing. We thus monitored the impact of these chemical inhibitors on formation of the Cot-1 hole at day 5 of XIST induction and observed it to be remarkably robust to all treatments, with no treatment showing a statistically significant weakening of depletion (Fig. 3C and D, Additional file 1: Table S6). Overall, we observed that the establishment of a Cot-1 hole was not particularly sensitive to disruption of individual chromatin marks, while spread of genic silencing up to 17 Mb was particularly sensitive to reduction in PRC2 enzymatic activity. Distance-dependent effects were observed for KMT5A and HDAC inhibition. In contrast to recent literature in mouse cells, in these human somatic cells, the impact of HDAC inhibition was not specific to HDAC3 [10, 23]. We have previously identified dependencies of MacroH2A recruitment on PRC2 activity and SMCHD1 recruitment on PRC1, implicating considerable interactions between pathways establishing XIST-dependent heterochromatin.

### Interdependencies between XIST-induced chromatin-remodelling pathways

**Fig. 4:**
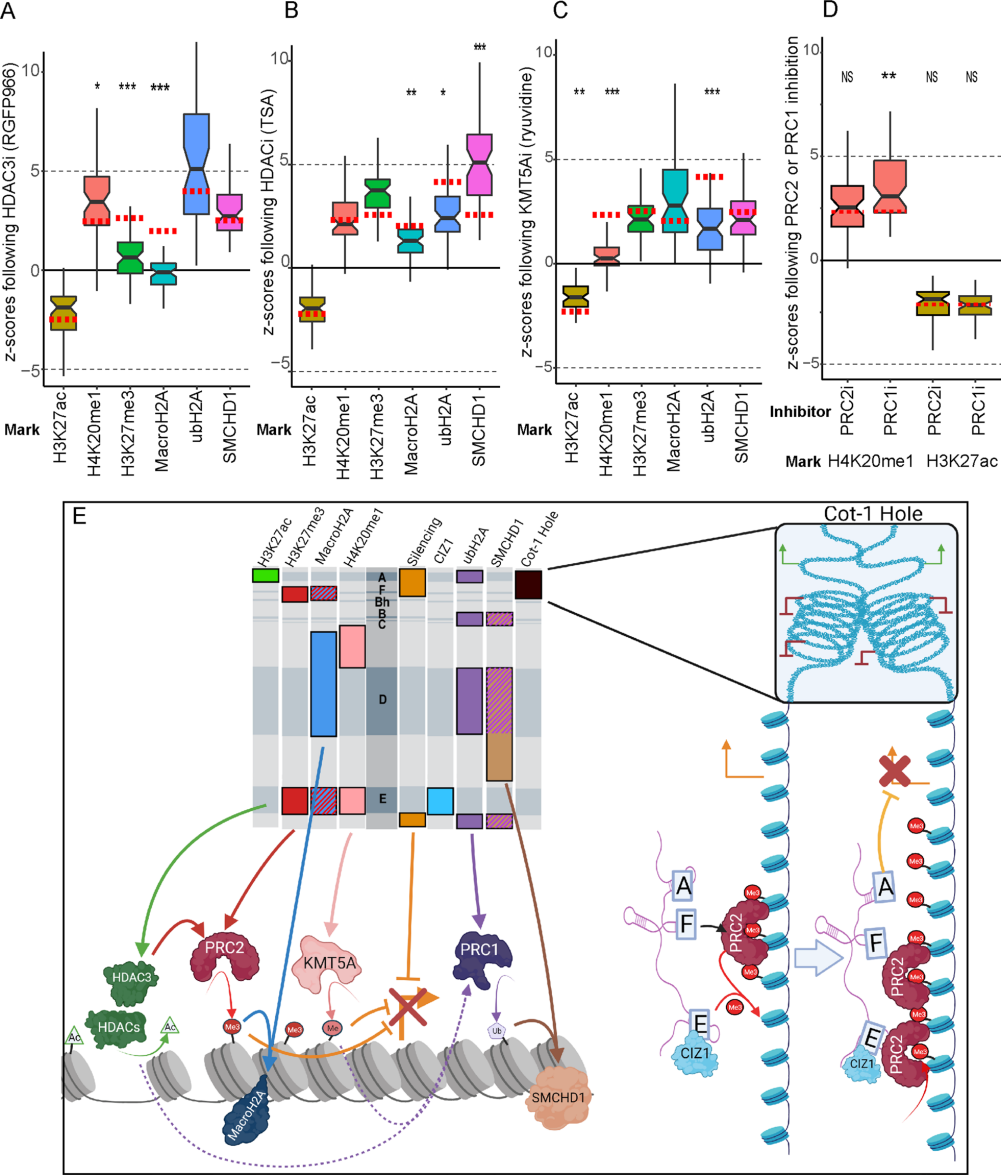
Examining the connections between XIST-dependent chromatin modifiers. **A**–**D** The relative distribution of chromatin features following chemical inhibition (59–61 cells each) are shown plotted according to the chemical inhibitor used. The median *z*-score of uninhibited Full XIST cells is shown as a dotted red line to provide a point of comparison. All conditions were blinded throughout the process and comparisons were made using the Mann–Whitney test with multiple testing correction (*p*-values * < 0.05, ** < 0.01, *** < 0.001). **A** Cells were treated with 1uM RGFP966 to inhibit HDAC3. **B** Cells were treated with 60 nM TSA to broadly inhibit HDACs. **C** Cells were treated with 4uM of ryuvidine to inhibit KMT5A. **D** Cells were treated with either PRC2 inhibitor GSK343 (5 µM) or PRC1 inhibitor PRT4165 (50 µM). **E** Summary of the functions of XIST domains with connections between the various chromatin-remodelling factors studied (created with BioRender.com). The hashed colours depict the dependence of MacroH2A on PRC2 activity and SMCHD1 on PRC1. Pointed arrows indicate activation or contribution to a process, flat-headed arrows indicate inhibition. Solid arrows indicate an essential role while dotted arrows indicate effects that are only modestly contributing to a process. The depiction on the right shows our model of how the A, F and E repeats contribute to gene silencing and the spread of H3K27me3. The Repeat F region is essential for aggregation to repressed domains, including pre-existing H3K27me3 regions. This brings Repeat A into proximity of expressed genes (resulting in their silencing); and brings Repeat E (and bound proteins like CIZ1) together to facilitate spread of H3K27me3 along the chromosome. However, Repeat E is not necessary for silencing, and Repeat A is not necessary for H3K27me3 recruitment. Repeat E facilitates the activation and/or recruitment of PRC2 to promote the spread of H3K27me3

In order to refine potential pathways through which XIST modifies the chromatin and epigenetic profile of its chromosome of origin, we set out to identify how the various chromatin-modifying complexes affect one another. Maximal levels of enrichment for most heterochromatin features required 5 days of XIST induction as described previously (Fig. 2A–C, [13]), with only MacroH2A still accumulating at day 5. To examine what role the catalytic activity of the various chromatin-modifying factors analysed so far had on overall chromatin architecture, we incubated cells with a combination of doxycycline and chemical inhibitors as described and performed IF-FISH analysis on populations of 60 cells. Inhibition of HDAC3 in the HT1080 Full XIST cells with 1uM of RGFP966 surprisingly showed no significant impact on the *z*-score of depletion of H3K27ac under the XIST signal (*z*-score = − 1.87, *p* = 0.42, Fig. 4A), despite raising the genome average levels of acetylation as monitored by western blotting ~ 2.9-fold (with H4K8ac; Additional file 1: Table S7). This suggests that the balance of depletion at XIST keeps pace with gains in acetylation. Inhibition of HDAC3, however, dramatically affected the enrichment of H3K27me3 (*z*-score = 0.63, *p* = 1.7 × 10^–11^), simultaneously reducing H3K27me3 genome-wide by 50% as seen by western (Additional file 7: Fig. S6C). Further, this treatment completely prevented MacroH2A enrichment (*z*-score = − 0.11, *p* = 6.1 × 10^–18^, Fig. 4A), which we had previously shown to be dependent on H3K27me3. Inhibition of HDAC3 resulted in a subtle rise in the average enrichment of H4K20me1 at the site of XIST (*z*-score = 3.44, *p* = 8.19 × 10^–4^) indicating that HDAC3 activity clearly is not essential for H4K20me1 enrichment. Inhibition of HDAC3 had no discernable negative effect on the enrichment of ubH2A and SMCHD1 (Fig. 4A).

We next set out to compare what effect broad-spectrum inhibition of HDAC activity would have on XIST activity using the chemical inhibitor TSA. We tested the ability of XIST to remodel its chromatin in the highest tolerable concentration of TSA (60 nM) over 5 days while XIST was being induced with doxycycline. As observed with HDAC3-specific inhibition, broad-spectrum HDAC inhibition had no effect on the relative depletion of H3K27ac at the XIST RNA cloud (*z*-score = − 2.0, *p* = 0.199) despite a roughly fourfold increase in global histone acetylation (Fig. 4B, Additional file 7: Fig. S6A). Broad spectrum inhibition of HDAC activity did not discernibly effect either H4K20me1 or H3K27me3 enrichment, however we did observe a decrease in the magnitude of MacroH2A enrichment (*z*-score  = 1.3, *p* = 3.51 × 10^–4^) and ubH2A (*z*-score = 2.4, *p* = 2.66 × 10^–3^) compared to the uninhibited control treatments (Fig. 4B and Additional file 1: Table S7). Interestingly, the drop in MacroH2A observed by both HDAC3i and broad HDACi was not observed with a similar decrease in H3K27me3 directly caused by 2.5uM of the PRC2-specific inhibitor GSK343 (Fig. 4A and B, [13]). A significant and unexpected increase in SMCHD1 enrichment was observed at the XIST RNA cloud (*z*-score = 5.1, *p* = 2.53 × 10^–5^, Fig. 4B). While beyond the scope of the current study, it is possible that the increased levels of global histone acetylation caused by broad HDAC inhibition resulted in a disproportionate decrease in SMCHD1 occupancy across the nucleus compared to the XIST loci.

As inhibition of KMT5A catalytic activity had a distance-dependent effect on silencing (Fig. 3A), yet no overlap with the domains needed for silencing, we examined the role of KMT5A on chromatin remodelling using the 4uM dose of ryuvidine found to dramatically reduce global H4K20me1 levels (Additional file 7: Fig. S6B). As anticipated, KMT5A inhibition effectively prevented any signs of H4K20me1 enrichment at the XIST RNA cloud (*z*-score = 0.26, *p* = 3.2 × 10^–14^, Fig. 4C) indicating that KMT5A activity was essential for the enrichment of H4K20me1. An effect of this inhibition was observed in the weakening of H3K27ac depletion at the site of XIST (*z*-score = − 1.6, *p* = 3.2 × 10^–4^, Fig. 4C, Additional file 1: Table S7). Inhibition of H4K20me1 produced a statistically significant weakening of the enrichment of ubH2A but did not completely abolish enrichment (*z*-score  = 1.68, *p* = 1.5 × 10^–8^, Fig. 4C). Taken together these results suggest that KMT5A-deposited H4K20me1 operates through mechanisms primarily independent of the other factors examined here.

Previous studies examining the role of PRC2 and PRC1 on XIST activity in this model system found that these two complexes operate independently, but are each critical for the recruitment of subsequent heterochromatin factors MacroH2A and SMCHD1, respectively [13]. We set out to identify whether the inhibition of either of these polycomb complexes affected the enrichment of H4K20me1 or the deacetylation of chromatin at the XIST RNA cloud. We performed IF-FISH for H4K20me1 and H3K27ac, respectively, in treatments with either 5uM of PRC2 inhibitor GSK343 or 50 µM of PRC1 inhibitor. Neither treatment weakened the enrichment of H4K20me1 or weakened the depletion of H3K27ac at the XIST RNA cloud (Fig. 4D and Additional file 1: Table S7). Inhibition of PRC1 resulted in a moderate increase in the enrichment of H4K20me1 (*z*-score = 3.07, *p* = 3.1 × 10^–4^). These results suggest that the role of polycomb complexes is dispensable for histone deacetylation and H4K20 monomethylation. Enrichment of CIZ1 at the XIST RNA was also observed to be recruited independently of any of the chromatin modifiers examined here, supporting the idea that its association is not dependent upon chromatin remodelling (Additional file 10: Fig. S9).

## Discussion

The XIST lncRNA is unique in its ability to initiate the establishment of an inactive chromosome, and remarkably induction of an XIST cDNA in human somatic cells is able to recapitulate silencing and establish many of the heterochromatic features of the inactive X. While mouse embryonic stem cells are reported to have a limited developmental window during which Xist induces inactivation (reviewed in 3), we have previously demonstrated that both these HT1080-fibrosarcoma derived cells as well as the near triploid 293 cells were able to induce silencing and many features of an inactive X [17]. These transformed cells may be more epigenetically pliable than other differentiated human cells; however, the HT1080 cells studied here are diploid with minimal chromosome rearrangements [16]. In this manuscript we utilize a series of 9 transgene deletions that span the XIST cDNA to determine the regions of XIST critical for silencing and chromatin remodelling. Growing evidence from research groups studying XIST, including our own, have suggested that interactions between domains of XIST RNA (both intramolecular and intermolecular) are not only endemic [8] but essential to numerous functions of XIST including chromatin remodelling [13, 24]. Deletions can not only remove domains of interacting proteins, but may also impact the RNA structure of the remaining RNA. Thus, in addition to examining the timing and domains required, we further followed the impact of inhibiting chromatin-remodelling pathways to examine interactions between pathways in silencing and identify critical and supporting players in XIST-mediated silencing. A summary of these results is shown in Fig. 4E. In these human cancer-derived somatic cells we establish that silencing of four genes, which are genes up to 17 Mb from the XIST integration site, occurs rapidly, being nearly complete within 2 days of dox induction of the Full XIST cDNA. The importance of the A repeat region for silencing was previously shown using this model system [17] as well as mouse [25]; while the A repeat region alone was shown to silence a proximal reporter gene next to the XIST construct [7]. We now confirm the importance of the A repeats in silencing, but demonstrate that two additional domains are also required, the ~ 1 kb including the Repeat F region and a 630-bp region at the non-repeat 3’ end of XIST (see Fig. 1B) for silencing. The requirement for the F repeat region is shared with recruitment of H3K27me3 [13], the mark deposited by PRC2 (see Fig. 4E), and PRC2 inhibition by GSK343 also impacted silencing in a dose-dependent fashion. The enrichment of the XIST RNA-coated chromatin domain with H3K27me3 further depended on the presence of the Repeat E region—but that region was not required for silencing. This relationship highlights the complexity and interrelatedness of the silencing process. We propose that the multiple regions of XIST critical for H3K27me3 enrichment regulate distinct steps in the process. As the ~ 1-kb region encompassing Repeat F is critical for silencing, Cot-1 hole formation and H3K27me3 enrichment, this region reflects a shared step in all these processes. A potential explanation is that the Repeat F region brings XIST to pre-existing sites of H3K27me3; coalescing those domains to initiate establishment of the Cot-1 hole and providing initial targeting/tethering sites for the XIST RNA. Once it is tethered to the chromosome, Repeat A acts to inactivate nearby genes thus creating the visible Cot-1 deletion; and further acts to recruit histone deacetylases. While Repeats A and F were comparable in their effect on the silencing of specific genes, we observed a statistically significant difference between their effect on Cot-1 hole formation, with loss of Delta F having the stronger effect. This difference may reflect the importance of Delta F not only in silencing genes, but also in the aggregation of chromatin into a silenced domain. These conclusions about the role of the region around Repeat F would then suggest that the role of Repeat E is to promote the spread of H3K27me3, either through the recruitment of PRC2 or through the activation of PRC2 already present at the chromatin, thereby establishing a visible enrichment of H3K27me3 under the XIST domain. All experiments supported our previous observation that MacroH2A enrichment at the XIST RNA cloud was dependent upon the enrichment of H3K27me3.

The Repeat E region of XIST seems to represent one of the most critical elements of the RNA, being essential for CIZ1 recruitment as well as the enrichment of H4K20me1, H3K27me3 and MacroH2A, although inhibition of KMT5A and PRC2, respectively, suggested that neither complex depends upon the catalytic activity of the other. Further, CIZ1 recruitment was not impacted by any of the inhibitors tested (Additional file 10: Fig. S9). Combined with CIZ1 recruitment being observable at every time point examined, this supports a direct interaction of CIZ1 with XIST at the E repeats, independent of silencing or chromatin remodelling. CIZ1 is reported to tether Xist to the nuclear matrix with reduction of CIZ1 resulting in a cell-type dependent failure of XIST to localize [15, 26]. In our cells, deletion of the E repeat did not delocalize XIST, similar to mouse early embryonic cells, that also demonstrate Xist-dependent silencing; which we observe with the HT1080 cells. Other work has demonstrated that multiple XIST-binding proteins (including PTBP1, MATR3, TDP-43 and CELF1) assemble at the multivalent E repeat regions resulting in formation of a condensate by self-aggregation and protein–protein interactions [21]. Such heterotypic protein interactions at the E repeat may contribute to the spread of H3K27me3 and H4K20me1.

The functional role of H4K20me1 still remains enigmatic, though our analysis of the domains critical for its enrichment as well as the effect of chemical inhibition on KMT5A provides several novel insights. Treatment of HT1080 cells with ryuvidine confirmed the role of KMT5A in the deposition of H4K20me1 to the inactive X. The cells demonstrated a striking tolerance for the loss of KMT5A activity, as after 5 days of treatment with the highest dose we saw nearly total loss of global H4K20me1 seen by western, despite the mark being critical during mouse development and involved in chromosome condensation [27]. In mouse cells, H4K20me1 was also recruited concurrently with H3K27me3, however, in contrast to our findings in these human somatic cells the recruitment was lost with a deletion of the B and C repeats [14]. Even with the near ablation of KMT5A activity by ryuvidine, there were only modest effects on XIST localization or other chromatin marks. At the highest level of KMT5A inhibition XIST was not able to silence the three most distal alleles examined after five days of induction. At the lower dose of KMT5A inhibition however, the strength of silencing at all four genes was still strong (> 70% of uninhibited levels) suggesting that silencing could still occur even when KMT5A activity was reduced by ~ 74%. Taken together these studies suggest that in human somatic cells the enrichment of H4K20me1 does not seem to depend upon other Xi associated heterochromatin features.

The A repeats were seen to be critical for silencing, formation of the Cot-1 hole, and also for histone deacetylation. While additional domains (F and 3’) were critical for silencing, only the A repeat region was required for histone deacetylation. The A repeats have been shown to bind multiple proteins, with a growing consensus that during mouse differentiation, the binding of SPEN activates HDAC3 through SMRT recruitment [9, 10]. Examining the impact of histone deacetylation in this model has revealed striking contrasts with the importance of HDACs, and particularly HDAC3, between model systems. In this human somatic cell model, we observe that HDAC3 inhibition by RGFP966 for 5 days showed no sign of XIST losing its ability to silence four distal genes. With 2 days of inhibition at even higher doses of RGFP966 only a minimal effect of silencing on SLC25A37 was detected. At the highest doses of the broad-spectrum HDAC inhibitor TSA, we did observe a statistically significant impact on the ability of XIST to silence distal genes. This effect appeared distance dependent, with the most distal genes SLC25A37 and STC1 (17 Mb away from XIST) remaining unsilenced while DLC1 (6 Mb away) was only partially silenced at the highest dose of TSA. Both TSA and RGFP966 produced comparable, significant dose-dependent increases in global histone acetylation detected by western. These findings demonstrate that XIST possesses HDAC3 independent silencing mechanisms, which has been suggested to exist in mice [23]. This was especially evident given that inhibition of HDAC3 in this model system effectively improved XIST-mediated silencing, though it is unclear what the reason for this is. Surprisingly, despite the dramatic increase in histone acetylation levels following 5 days of either TSA or RGFP966 treatment, the impact of either treatment on the ability of XIST to create an acetylation-depleted chromatin territory was minimal. It may be that XIST is able to exclude euchromatin territories from its compartment in the nucleus, and that this contributed to the relative depletion of histone acetylation that we observed. While HDAC3 catalytic activity did not appear to impact gene silencing, it was essential for the enrichment of H3K27me3 and MacroH2A. While this finding is novel in the field of XCI and XIST, there have been studies suggesting that HDAC3 can recruit PRC2 to regulate specific regions of the genome and it may be that in this context HDAC3 catalytic activity is the crucial factor necessary for the spread of H3K27me3 [28]. Interestingly, broad-spectrum inhibition of TSA seemed to have no significant impact on H3K27me3, a moderate reduction in macroH2A enrichment and actually increased SMCHD1 occupancy at XIST relative to the nuclear background. It is unclear why this increase in the enrichment of the heterochromatin-associated SMCHD1 protein occurred given the global rise in euchromatin associated histone acetylation globally, though one explanation could be that lower levels of SMCHD1 occupancy across the nucleus could have inflated the standard score if occupancy was unchanged at the XIST RNA cloud.

Inhibition of PRC1 had no negative effect on XIST-mediated gene silencing and did not attenuate Cot-1 hole formation (in fact Cot-1 holes were more distinct following PRC1 inhibition). It may be that enrichment of ubH2A or its accumulation across the XIST bound chromatin depends upon a multifactorial set of changes in the chromatin. It has been shown in mouse models that hnRNPK is crucial for PRC1 recruitment and the human Repeat B, C and D regions contribute to this recruitment of PRC1 via hnRNPK binding [8, 29]. The importance of the repeat A and the 3’ region of XIST for the enrichment of ubH2A may represent a dependency on these regions’ abilities to induce gene silencing, as studies in mouse models have supported the idea that the spread of ubH2A is dependent upon Xist-mediated gene silencing [23]. The contributing role of HDAC and KMT5A activity for complete ubH2A enrichment by XIST could be due to these factors contributing to the spread of ubH2A or affecting the association/activity of PRC1 with XIST-associated sequences. In this model system, however, it appears that ubH2A enrichment represents a relatively downstream process, as inhibition of PRC1 activity has been shown to have no negative impact of XIST-mediated heterochromatin formation, apart from the recruitment of SMCHD1 as described previously [13].

## Conclusions

To examine the role of human XIST in silencing of a chromosome we utilize a model system in which nine deletions of a full-length XIST cDNA transgene on an autosome can be induced and followed for both silencing and chromatin changes. In contrast to studies in mouse developmental models, we find that silencing relies not only upon Repeat A, but also requires two additional regions, the Repeat F encompassing region and the terminal 3’ end of the cDNA. Genic silencing was found to be reliant on the activity of chromatin-remodelling factors, including PRC2 and to a lesser extent KMT5A. The formation of a Cot-1 transcriptionally inert domain, however, was found to be highly resistant to perturbations of individual chromatin-remodelling complexes as well as deletion of the 3’ region of XIST, suggesting that this characteristic feature of XIST is a result of multiple independent and redundant processes. Histone deacetylase activity required only the Repeat A region, yet in contrast to previous studies, it appeared that HDAC3 influenced PRC2 while other HDACs influenced silencing. We demonstrate that the regions of XIST examined often possess multiple independent activities, as exemplified by the role of repeat E for the recruitment of PRC2, KMT5A and CIZ1, all of which were found to operate independently. The enrichment of H4K20me1 at the XIST RNA cloud by KMT5A was shown to depend upon multiple independent and interdependent domains, a common finding in our model system. H4K20me1 was found to operate without dependency on any of the other chromatin-modifying factors examined, but was found to have a contributing role to the spread of gene silencing and the enrichment of ubH2A. Overall, this study provides a map of the connection between the key heterochromatin features established by XIST as well as the ability of XIST to induce gene silencing.

## Supplementary Information

**Additional file 1: Tables S1–S8.** **Table S1**: Timepoints for FISH and IF-FISH analysis. List of the factors analyzed in the Full XIST 8p HT1080 cell line over the course of multiple days. The factors analyzed either by FISH in the case of Cot-1 or IF in the case of the protein/chromatin are shown in the first column. The second column lists the period of time XIST was induced. The number of cells analyzed, the mean and median *z*-score calculated as well as the standard deviation (SD) for each condition are listed. The statistical significance of each time point relative to each other were calculated using the Mann–Whitney *U* test and the p values are listed. **Table S2.** Summary of Deletion Constructs. Each of the cell lines successfully generated for each type of deletion construct are listed along with the gRNA targeting sequence used to create the deletion and the total number of nucleotides lost from the XIST cDNA sequence. **Table S3.** Deletion constructs ability to form Cot-1 hole. Probes for Cot-1 were used to measure the formation of a Cot-1 hole by inducible XIST constructs in 8p. The constructs tested are listed in the second column followed by the cell type, number of cells analyzed, the mean and median *z*-score calculated as well as the standard deviation (SD) for each construct. The statistical significance of each population of deletion constructs’ difference from Full XIST in their *z*-score s was calculated using the Mann–Whitney U test and the p values are listed. **Table S4.** The ability of the deletion constructs to remodel chromatin and recruit CIZ1. List of the factors analyzed, cell type, number of cells analyzed, the mean and median *z*-score calculated as well as the standard deviation (SD) for each construct. The statistical significance of each population of deletion constructs’ difference from Full XIST in their enrichment was calculated using the Mann–Whitney U test and the p values are listed. **Table S5.** Summary of effect of chemical inhibitors on XIST mediated silencing. The average strength of silencing along with statistical significance of the chemical inhibitor treatments for each allele tested in this paper are shown below. The upper section of the table shows the average strength of silencing and *p*-values calculated for each condition relative to 5ddox treated Full XIST described in Fig. 3A. The bottom section shows the average strength of silencing and *p*-values calculated relative to 2ddox treated Full XIST (Fig. 3B). *p*-values were calculated by *t*-test. The threshold of statistical significance in this paper was adjusted using the Bonferroni correction. **Table S6.** Effect of chemical inhibitors on XIST formation of Cot-1 holes. The effect of chemical inhibition for 5 days along with the induction of Full XIST on the formation of Cot-1 holes are shown in this table. The chemical inhibitors and doses used for each test is shown in the first column. The number of cells analyzed, the mean, median and standard deviation of *z*-score s for each condition are shown in the subsequent columns. The statistical significance of each population of deletion constructs’ difference from induced Full XIST untreated with chemical inhibitors was calculated using the Mann–Whitney U test and the p values are listed. **Table S7.** Effect of chemical inhibition treatment on XIST chromatin remodelling. The effect of chemical inhibition for 5 days along with the induction of Full XIST on the chromatin remodelling are shown in this table. The chemical inhibitors and doses used for each test is shown in the first column. The number of cells analyzed, the mean, median and standard deviation of *z*-score s for each condition are shown in the subsequent columns. The statistical significance of each population of deletion constructs’ difference from induced Full XIST untreated with chemical inhibitors was calculated using the Mann–Whitney U test and the p values are listed. **Table S8.** list of primers used throughout this experiment. The names of the various primers used throughout the experiments described. Primers were labeled either F (forward) or R (reverse) to denote their orientation during amplification. All sequences are orientated 5’ to 3’. **Table S9.** Table of antibodies used for IF and western blotting.

**Additional file 2: Fig. S1.** Original XIST deletion constructs confirm importance of 3’ region for silencing of transcription. The ability of the original deletion constructs (Delta PflMI, Exon 1 and Delta Delta) to silence four distal genes. Strength of silencing was normalized to Full XIST. Three biological replicates were tested for each condition shown and statistical significance of a treatment’s effect on XIST induced silencing was calculated using a *t*-test with multiple testing correction and those constructs where silencing of all four genes were impacted are shown (*p < 0.05).

**Additional file 3: Fig. S2.** Example images of Cot-1 depletion in XIST deletion constructs. Example IF-FISH images of the nuclei of HT1080 XIST CRISPR deletion constructs following 5 days of dox induction. The top three photos for each type of deletion construct are the merged colour channels showing DAPI (blue), XIST RNA (green) and Cot-1 (red). Below each merged channel is the single channel of XIST RNA (green) and Cot-1 red. All images are squares with each side ranging from 24 to 27 µm in length.

**Additional file 4: Fig. S3.** Example images of H3K27ac depletion in XIST deletion constructs. Example IF-FISH images of the nuclei of HT1080 XIST CRISPR deletion constructs following 5 days of dox induction. The top three photos for each type of deletion construct are the merged colour channels showing DAPI (blue), XIST RNA (green) and H3K27ac (red). Below each merged channel is the single channel of XIST RNA (green) and H3K27ac (red). All images are squares with each side ranging from 24 to 27 µm in length.

**Additional file 5: Fig. S4.** Example images of H4K20me1 enrichment in XIST deletion constructs. Example IF-FISH images of the nuclei of HT1080 XIST CRISPR deletion constructs following 5 days of dox induction. The top three photos for each type of deletion construct are the merged colour channels showing DAPI (blue), XIST RNA (green) and H4K20me1 (red). Below each merged channel is the single channel of XIST RNA (green) and H4K20me1 (red). All images are squares with each side ranging from 24 to 27 µm in length.

**Additional file 6: Fig. S5.** Example images of CIZ1 depletion in XIST deletion constructs. Example IF-FISH images of the nuclei of HT1080 XIST CRISPR deletion constructs following 5 days of dox induction. The top three photos for each type of deletion construct are the merged colour channels showing DAPI (blue), XIST RNA (green) and CIZ1 (red). Below each merged channel is the single channel of XIST RNA (green) and CIZ1 (red). All images are squares with each side ranging from 24 to 27 µm in length.

**Additional file 7: Fig. S6.** Western blots showing effect of chemical inhibitors on global histone modification levels. Western blotting images demonstrating the levels of modified histone marks following treatment of HT1080 cells with chemical inhibitors. The bands for each chromatin feature as well as endogenous control are shown and the computed relative levels of each chromatin mark relative to the uninhibited controls are shown underneath the bands. A) Levels of histone acetylation (H4K8ac) following HDAC treatment as well as treatment with GSK343. B) Levels of H4K20me1 following Ryuvidine treatments C) Effect of GSK343 inhibition on H3K27me3 levels, in addition to HDAC inhibitors. D) Levels of ubH2A following PRC1 inhibition with PRT4165. C-D) Versions of these western blots were published previously in our investigation of the effect of GSK343 and PRT4165 on HT1080 cells [13]. The inhibitors used to target the activity of each factor are: TSA, broad spectrum HDACs; RGFP966, HDAC3; ryuvidine, PRSET7; GSK343, PRC2; PRT4165, PRC1.

**Additional file 8: Fig. S7.** Allelic contribution unaffected by HDAC3 inhibition in absence of XIST. The allelic contribution of the four SNP coding alleles is shown as measured by pyrosequencing. Allelic contribution to the expression of each gene is shown in the presence or absence of HDAC3 inhibitor, RGFP966, for five days with or without the induced expression of XIST with dox. Statistical significance was calculated using a *t*-test with thresholds adjusted for multiple testing correction from * < 0.05, ** < 0.01, *** < 0.001.

**Additional file 9: Fig. S8.** Effect of chemical inhibitors on XIST RNA levels. Relative expression of XIST RNA following treatment of HT1080 cells with chemical inhibitors. Individual dots for each treatment condition show the relative levels of biological replicates compared to the average levels of time matched dox treated cells. Differences in XIST levels in any of the treatment conditions were calculated using *t*-test with multiple testing correction (*p < 0.05). A) Relative levels of XIST following five days of induction with 1ug/ml dox and the chemical inhibitor listed. B) Relative levels of XIST following two days of induction with 1ug/ml dox and the chemical inhibitor listed.

**Additional file 10: Fig. S9.** Effect of chemical inhibition on the enrichment of CIZ1 at XIST RNA. The relative levels of CIZ1 (*z*-score) at the site of XIST RNA in the HT1080 cell lines following five days of chemical inhibition treatment. Populations of 60 cells were analyzed per condition and were compared by Mann–Whitney test to an uninhibited control population of HT1080 cells, all of which had Full XIST induced for 5 days. None of the conditions differed significantly from the control.

## Data Availability

All data generated or analysed during this study are included in this published article [and its supplementary information files].

## Abbreviations

5ddox: 5 Days of doxycycline treatment
CRISPR: Clustered regulatory interspaced short palindromic repeats
DMEM: Dulbecco's modified Eagle medium
FISH: Fluorescent in situ hybridization
HDAC: Histone deacetylase
IF: Immunofluorescence
LncRNA: Long non-coding RNA
PRC: Polycomb repressive complex
Rt-qPCR: Reverse transcribed quantitative polymerase chain reaction
UbH2A: H2AK119ub
