# Supplementary material for: Multiple distinct domains of human XIST are required to coordinate gene silencing and subsequent heterochromatin formation
Source: Epigenetics Chromatin. 2022 Feb 4;15:6. doi: 10.1186/s13072-022-00438-7 (PMC8815261; doi:10.1186/s13072-022-00438-7)
Supplement: Supplementary file 1 — Additional file 1: Tables S1–S8. Table S1: Timepoints for FISH and IF-FISH analysis. List of the factors analyzed in the Full XIST 8p HT1080 cell line over the course of multiple days. The factors analyzed either by FISH in the case of Cot-1 or IF in the case of the protein/chromatin are shown in the first column. The second column lists the period of time XIST was induced. The number of cells analyzed, the mean and median z-score calculated as well as the standard deviation (SD) for each condition are listed. The statistical significance of each time point relative to each other were calculated using the Mann–Whitney U test and the p values are listed. Table S2. Summary of Deletion Constructs. Each of the cell lines successfully generated for each type of deletion construct are listed along with the gRNA targeting sequence used to create the deletion and the total number of nucleotides lost from the XIST cDNA sequence. Table S3. Deletion constructs ability to form Cot-1 hole. Probes for Cot-1 were used to measure the formation of a Cot-1 hole by inducible XIST constructs in 8p. The constructs tested are listed in the second column followed by the cell type, number of cells analyzed, the mean and median z-score calculated as well as the standard deviation (SD) for each construct. The statistical significance of each population of deletion constructs’ difference from Full XIST in their z-score s was calculated using the Mann–Whitney U test and the p values are listed. Table S4. The ability of the deletion constructs to remodel chromatin and recruit CIZ1. List of the factors analyzed, cell type, number of cells analyzed, the mean and median z-score calculated as well as the standard deviation (SD) for each construct. The statistical significance of each population of deletion constructs’ difference from Full XIST in their enrichment was calculated using the Mann–Whitney U test and the p values are listed. Table S5. Summary of effect of chemical inhibitors on XIST medi [file 13072_2022_438_MOESM1_ESM.docx]

**Table S1: Timepoints for FISH and IF-FISH analysis**

List of the factor analyzed in the Full XIST 8p HT1080 cell line over the course of multiple days. The factor analyzed either by FISH in the case of Cot-1 or IF in the case of the protein/chromatin are shown in the first column. The second column lists period of time XIST was induced. The number of cells analyzed, the mean and median z-score calculated as well as the standard deviation (SD) for each condition are listed. The statistical significance of each time point relative to each other were calculated using the Mann-Whitney U test and the p values are listed.

| **Mark** | **Days of Induction** | **# of cells** | **Mean**  **(z-score)** | **Median**  **(z-score)** | **S.D.**  **(z-score)** | **p-value to 2 day** | **p-value to 5 day** |
| --- | --- | --- | --- | --- | --- | --- | --- |
| Cot-1 | 2 days | 60 | -1.070 | -1.062 | 0.687 | -- | -- |
| Cot-1 | 5 days | 60 | -1.407 | -1.412 | 0.609 | 9.45E-03 | -- |
| Cot-1 | 10 days | 60 | -1.517 | -1.411 | 0.624 | 4.87E-04 | 4.23E-01 |
| H3K27ac | 2 days | 60 | -1.522 | -1.618 | 0.799 | -- | -- |
| H3K27ac | 5 days | 60 | -2.259 | -2.163 | 1.054 | 7.83E-05 | -- |
| H3K27ac | 10 days | 61 | -2.276 | -2.172 | 1.088 | 7.39E-05 | 9.98E-01 |
| H4K20me1 | 2 days | 61 | 1.577 | 1.433 | 1.640 | -- | -- |
| H4K20me1 | 5 days | 61 | 2.432 | 2.489 | 1.594 | 1.01E-03 | -- |
| H4K20me1 | 10 days | 60 | 3.033 | 2.531 | 1.780 | 3.44E-06 | 1.07E-01 |
| CIZ1 | 2 days | 63 | 14.762 | 12.808 | 6.856 | -- | -- |
| CIZ1 | 5 days | 60 | 11.753 | 10.972 | 5.569 | 1.40E-02 | -- |
| CIZ1 | 10 days | 60 | 13.344 | 12.731 | 6.194 | 3.51E-01 | 1.59E-01 |

**Table S2: Summary of Deletion Constructs**

Each of the cell lines successfully generated for each type of deletion construct are listed along with the gRNA targeting sequence used to create the deletion and the total number of nucleotides lost from the XIST cDNA sequence.

| **Deletion cell line** | **5’ gRNA target sequence** | **3’ gRNA target sequence** | **Nucleotides deleted** |
| --- | --- | --- | --- |
| Δ A #4 | GGACGTGTCAAGAAGACACT | GTTTGTGCTAAGTTAAACTA | 776 |
| Δ A #12 | GGACGTGTCAAGAAGACACT | GTTTGTGCTAAGTTAAACTA | 777 |
| Δ A #22 | GGACGTGTCAAGAAGACACT | GTTTGTGCTAAGTTAAACTA | 776 |
| Δ FBh #21 | GTTTGTGCTAAGTTAAACTA | GGGGAGGTATACTTAGCCTT | 1127 |
| Δ FBh #22 | GTTAAACTAGGGAGGCAAGA | GGGGAGGTATACTTAGCCTT | 811 |
| Δ Bh #5 | GCAGCTGTCTTTAGCCAGTC | GAGTGTTTGAAGGTTTACAC | 833 |
| Δ Bh #7 | GCAGCTGTCTTTAGCCAGTC | GAGTGTTTGAAGGTTTACAC | 833 |
| Δ Bh #11 | GCAGCTGTCTTTAGCCAGTC | GAGTGTTTGAAGGTTTACAC | 857 |
| Δ BC #2 | GATGATCGTTGGCCAACAGG | GAGTGCTGTCTAATCCAAT | 1195 |
| Δ BC #8 | GATGATCGTTGGCCAACAGG | GAGTGCTGTCTAATCCAAT | 1189 |
| Δ BC #17 | GATGATCGTTGGCCAACAGG | GAGTGCTGTCTAATCCAAT | 1195 |
| Δ 3’PflMI #3 | GGACAAAGAATTTCCTTACT | GGCCAAGAAATGGGGCCTT | 2859 |
| Δ 3’PflMI #6 | GGACAAAGAATTTCCTTACT | GGCCAAGAAATGGGGCCTT | 2859 |
| Δ D #3 | GCAGTAATGCAAATGGAGCA | GCCAAGAAAAGGGGACTTAG | 3084 |
| Δ D #10 | GCAGTAATGCAAATGGAGCA | GCCAAGAAAAGGGGACTTAG | 3092 |
| Δ 3D5E #13 | GCCAAGAAAAGGGGACTTAG | GTGAAAGAAGAGCCACATCT | 3584 |
| Δ 3D5E #14 | GCCAAGAAAAGGGGACTTAG | GTGAAAGAAGAGCCACATCT | 3583 |
| Δ 3D5E #15 | GCCAAGAAAAGGGGACTTAG | GTGAAAGAAGAGCCACATCT | 3588 |
| Δ E #6 | GCCTGGCACTCTAGCACTTG | GTTGGGGAAAAAAAAGTGCC | 1844 |
| Δ E #10 | GCCTGGCACTCTAGCACTTG | GTTGGGGAAAAAAAAGTGCC | 1778 |
| Δ 3’ #1 | GTTGGGGAAAAAAAAGTGCC | GTCACAATTGAAACAAACTG | 630 |
| Δ 3’ #7 | GTTGGGGAAAAAAAAGTGCC | GTCACAATTGAAACAAACTG | 630 |

**Table S3: Deletion constructs ability to form Cot-1 hole.**

Probes for Cot-1 were used to measure the formation of a Cot-1 hole by inducible XIST constructs in 8p. The construct tested are listed in the second column followed by the cell type, number of cells analyzed, the mean and median z-score calculated as well as the standard deviation (SD) for each construct. The statistical significance of each population of deletion constructs’ difference from Full XIST in their z-scores was calculated using the Mann-Whitney U test and the p values are listed.

| **Probe** | **Construct** | **# of cells** | **Mean**  **(z-score)** | **Median**  **(z-score)** | **S.D.**  **(z-score)** | **p-value to control** |
| --- | --- | --- | --- | --- | --- | --- |
| Cot-1 | Fulll XIST | 60 | -1.407 | -1.412 | 0.609 | N/A |
| Cot-1 | Δ A | 60 | -0.431 | -0.483 | 0.613 | 6.57E-13 |
| Cot-1 | Δ FBh | 61 | 0.160 | 0.182 | 0.910 | 1.47E-16 |
| Cot-1 | Δ Bh | 60 | -1.547 | -1.513 | 0.625 | 2.13E-01 |
| Cot-1 | Δ BC | 60 | -1.454 | -1.344 | 0.759 | 9.18E-01 |
| Cot-1 | Δ 3’PflMI | 60 | -1.332 | -1.308 | 0.498 | 6.84E-01 |
| Cot-1 | Δ D | 60 | -1.464 | -1.524 | 0.586 | 4.51E-01 |
| Cot-1 | Δ3’D5’E | 60 | -1.529 | -1.368 | 0.647 | 3.79E-01 |
| Cot-1 | Δ E | 61 | -1.034 | -1.071 | 1.007 | 2.21E-02 |
| Cot-1 | Δ 3’ | 60 | -1.401 | -1.430 | 0.651 | 7.91E-01 |

**Table S4: The ability of the deletion constructs to remodel chromatin and recruit CIZ1**

List of the factor analyzed, cell type, number of cells analyzed, the mean and median z-score calculated as well as the standard deviation (SD) for each construct. The statistical significance of each population of deletion constructs’ difference from Full XIST in their enrichment was calculated using the Mann-Whitney U test and the p values are listed.

| **Mark** | **Construct** | **# of cells** | **Mean**  **(z-score)** | **Median**  **(z-score)** | **S.D.**  **(z-score)** | **p-value to control** |
| --- | --- | --- | --- | --- | --- | --- |
| H3K27ac | Fulll XIST | 60 | -2.259 | -2.163 | 1.054 | N/A |
| H3K27ac | Δ A | 60 | -0.012 | -0.169 | 0.988 | 2.64E-18 |
| H3K27ac | Δ FBh | 59 | -1.855 | -1.754 | 1.085 | 4.77E-02 |
| H3K27ac | Δ Bh | 60 | -2.229 | -2.197 | 0.830 | 9.60E-01 |
| H3K27ac | Δ BC | 60 | -2.102 | -1.904 | 0.873 | 2.58E-01 |
| H3K27ac | Δ 3’PflMI | 60 | -2.337 | -2.199 | 1.069 | 7.67E-01 |
| H3K27ac | Δ D | 60 | -2.218 | -2.195 | 0.913 | 9.06E-01 |
| H3K27ac | Δ3’D5’E | 60 | -1.542 | -1.520 | 1.005 | 2.22E-04 |
| H3K27ac | Δ E | 60 | -2.144 | -2.039 | 0.758 | 5.31E-01 |
| H3K27ac | Δ 3’ | 60 | -1.993 | -1.936 | 1.409 | 1.10E-01 |
| H4K20me1 | Fulll XIST | 60 | 2.432 | 2.489 | 1.594 | N/A |
| H4K20me1 | Δ A | 60 | 2.187 | 2.052 | 1.327 | 2.57E-01 |
| H4K20me1 | Δ FBh | 60 | 2.290 | 2.191 | 1.187 | 5.84E-01 |
| H4K20me1 | Δ Bh | 60 | 2.018 | 1.827 | 1.097 | 1.07E-01 |
| H4K20me1 | Δ BC | 60 | 1.999 | 2.038 | 0.779 | 7.93E-02 |
| H4K20me1 | Δ 3’PflMI | 60 | 0.251 | 0.027 | 1.686 | 1.19E-10 |
| H4K20me1 | Δ D | 60 | 2.106 | 1.965 | 1.240 | 2.11E-01 |
| H4K20me1 | Δ3’D5’E | 60 | 2.158 | 2.080 | 1.143 | 2.89E-01 |
| H4K20me1 | Δ E | 60 | 0.404 | 0.432 | 0.845 | 3.44E-13 |
| H4K20me1 | Δ 3’ | 60 | 2.316 | 2.311 | 1.387 | 8.21E-01 |
| CIZ1 | Fulll XIST | 60 | 11.753 | 10.972 | 5.569 | N/A |
| CIZ1 | Δ A | 60 | 7.879 | 7.184 | 3.853 | 5.62E-05 |
| CIZ1 | Δ FBh | 60 | 12.073 | 11.717 | 4.138 | 3.82E-01 |
| CIZ1 | Δ Bh | 61 | 10.150 | 9.582 | 5.008 | 1.26E-01 |
| CIZ1 | Δ BC | 60 | 9.821 | 8.926 | 5.046 | 7.83E-02 |
| CIZ1 | Δ 3’PflMI | 61 | 13.275 | 12.598 | 6.066 | 1.35E-01 |
| CIZ1 | Δ D | 60 | 13.854 | 13.411 | 6.222 | 5.44E-02 |
| CIZ1 | Δ3’D5’E | 60 | 9.913 | 9.696 | 4.762 | 1.20E-01 |
| CIZ1 | Δ E | 60 | 0.109 | -0.028 | 0.872 | 3.56E-21 |
| CIZ1 | Δ 3’ | 60 | 13.623 | 12.668 | 7.499 | 2.88E-01 |

**Table S5: Summary of effect of chemical inhibitors on XIST-mediated silencing**

The average strength of silencing along with statistical significance of the chemical inhibitor treatments for each allele tested in this paper are shown below. The upper section of the table shows the average strength of silencing and p-values calculated for each condition relative to 5ddox treated Full XIST described in Figure 3A. The bottom section shows the average strength of silencing and p-values calculated relative to 2ddox treated Full XIST (Figure 3B). p-values were calculated by t-test. The threshold of statistical significance in this paper was adjusted using the Bonferroni correction.

|  | **CTSB average** | **p-value** | **DLC1 average** | **p-value** | **SLC25A37 average** | **p-value** | **STC1 average** | **p-value** |
| --- | --- | --- | --- | --- | --- | --- | --- | --- |
| **Ctrl** | 1.00 | 1.00E+00 | 1.00 | 1.00E+00 | 1.00 | 1.00E+00 | 1.00 | 1.00E+00 |
| **TSA 40nM** | 1.08 | 1.14E-02 | 1.00 | 9.11E-01 | 1.07 | 1.05E-02 | 1.03 | 7.18E-03 |
| **TSA 60nM** | 1.10 | 6.21E-03 | 1.01 | 7.05E-01 | 1.07 | 2.23E-02 | 1.03 | 3.49E-02 |
| **RGFP966 1uM** | 1.38 | 4.87E-07 | 1.06 | 2.11E-01 | 1.23 | 4.82E-05 | 1.22 | 5.41E-08 |
| **Ryuvidine 2 uM** | 0.95 | 3.35E-01 | 0.72 | 6.02E-05 | 0.81 | 2.13E-04 | 0.91 | 1.74E-04 |
| **Ryuvidine 4 uM** | 0.90 | 8.26E-03 | 0.30 | 1.99E-06 | 0.23 | 6.43E-08 | 0.25 | 6.33E-10 |
| **GSK 2.5uM** | 0.35 | 3.07E-07 | 0.54 | 3.78E-07 | 0.58 | 4.47E-07 | 0.60 | 1.57E-10 |
| **GSK343 5uM** | 0.16 | 1.71E-08 | 0.22 | 5.45E-08 | 0.14 | 9.94E-07 | 0.04 | 5.97E-04 |
| **PRT4165 25uM** | 0.92 | 6.45E-02 | 0.93 | 3.78E-02 | 0.85 | 1.51E-03 | 0.91 | 2.78E-04 |
| **PRT4165 50uM** | 0.94 | 2.34E-01 | 0.86 | 3.62E-03 | 0.86 | 3.56E-03 | 0.92 | 2.34E-04 |
| **RGFP966 2uM** | 1.01 | 9.11E-01 | 0.92 | 3.94E-02 | 0.72 | 2.23E-03 | 0.86 | 1.90E-04 |
| **RGFP966 5uM** | 1.16 | 1.18E-01 | 0.98 | 4.56E-01 | 0.80 | 7.92E-03 | 0.87 | 1.49E-03 |
| **RGFP966 10uM** | 1.08 | 3.66E-01 | 0.99 | 6.68E-01 | 0.74 | 3.26E-03 | 0.95 | 5.01E-02 |
| **TSA 100nM** | 1.06 | 3.37E-01 | 0.93 | 2.03E-02 | 0.59 | 1.75E-04 | 0.74 | 1.62E-05 |
| **TSA 200nM** | 1.06 | 6.90E-01 | 0.77 | 3.56E-04 | 0.17 | 6.99E-06 | 0.45 | 2.32E-06 |
| **TSA 300nM** | 0.80 | 8.51E-02 | 0.61 | 3.46E-04 | -0.01 | 8.87E-06 | 0.20 | 1.64E-06 |

**Table S6: Effect of chemical inhibitors on XIST formation of Cot-1 holes**

The effect of chemical inhibition for 5 days along with the induction of Full XIST on the formation of Cot-1 holes are shown in this table. The chemical inhibitors and doses used for each test is shown in the first column. The number of cells analyzed, the mean, median and standard deviation of z-scores for each condition are shown in the subsequent columns. The statistical significance of each population of deletion constructs’ difference from induced Full XIST untreated with chemical inhibitors was calculated using the Mann-Whitney U test and the p values are listed.

| **Treatment** | **# of cells** | **Mean**  **(z-score)** | **Median**  **(z-score)** | **S.D.**  **(z-score)** | **p-value to control** |
| --- | --- | --- | --- | --- | --- |
| Control | 60 | -1.407 | -1.412 | 0.609 | N/A |
| TSA 60nM | 60 | -1.329 | -1.212 | 0.582 | 3.60E-01 |
| RGFP966 1uM | 60 | -1.636 | -1.587 | 0.564 | 3.33E-02 |
| Ryuvidine 2uM | 60 | -1.279 | -1.151 | 0.843 | 2.43E-01 |
| Ryuvidine 4uM | 60 | -1.032 | -1.107 | 0.889 | 1.99E-02 |
| GSK343 5uM | 60 | -1.200 | -1.147 | 0.690 | 1.35E-01 |
| PRT4165 50uM | 60 | -1.859 | -1.849 | 0.711 | 5.48E-04 |

**Table S7: Effect of chemical inhibition treatment on XIST chromatin remodelling**

The effect of chemical inhibition for 5 days along with the induction of Full XIST on the chromatin remodelling are shown in this table. The chemical inhibitors and doses used for each test is shown in the first column. The number of cells analyzed, the mean, median and standard deviation of z-scores for each condition are shown in the subsequent columns. The statistical significance of each population of deletion constructs’ difference from induced Full XIST untreated with chemical inhibitors was calculated using the Mann-Whitney U test and the p values are listed.

| **Treatment** | **Factor** | **# of cells** | **Mean**  **(z-score)** | **Median**  **(z-score)** | **S.D.**  **(z-score)** | **p-value to untreated** |
| --- | --- | --- | --- | --- | --- | --- |
| N/A | H3K27ac | 60 | -2.259 | -2.163 | 1.054 | N/A |
| N/A | H4K20me1 | 61 | 2.432 | 2.489 | 1.594 | N/A |
| N/A | H3K27me3 | 60 | 2.901 | 2.593 | 1.704 | N/A |
| N/A | MacroH2A | 60 | 2.249 | 1.958 | 1.469 | N/A |
| N/A | ubH2A | 60 | 4.745 | 4.178 | 3.179 | N/A |
| N/A | SMCHD1 | 60 | 3.137 | 2.704 | 2.167 | N/A |
| RGFP966 1uM | H3K27ac | 59 | -2.178 | -1.870 | 1.407 | 4.21E-01 |
| RGFP966 1uM | H4K20me1 | 60 | 3.586 | 3.444 | 2.073 | 8.19E-04 |
| RGFP966 1uM | H3K27me3 | 60 | 0.749 | 0.634 | 1.245 | 1.74E-11 |
| RGFP966 1uM | MacroH2A | 60 | -0.150 | -0.113 | 0.762 | 6.06E-18 |
| RGFP966 1uM | ubH2A | 60 | 6.514 | 5.152 | 5.034 | 5.44E-02 |
| RGFP966 1uM | SMCHD1 | 60 | 2.829 | 2.734 | 1.184 | 7.39E-01 |
| TSA 60nM | H3K27ac | 60 | -1.968 | -1.945 | 0.951 | 1.99E-01 |
| TSA 60nM | H4K20me1 | 60 | 2.421 | 2.127 | 1.417 | 9.52E-01 |
| TSA 60nM | H3K27me3 | 60 | 3.725 | 3.744 | 1.354 | 3.21E-03 |
| TSA 60nM | MacroH2A | 60 | 1.459 | 1.305 | 1.369 | 3.51E-04 |
| TSA 60nM | ubH2A | 60 | 3.236 | 2.422 | 3.010 | 2.66E-03 |
| TSA 60nM | SMCHD1 | 60 | 5.124 | 5.084 | 2.658 | 2.53E-05 |
| Ryuvidine 4uM | H3K27ac | 60 | -1.670 | -1.612 | 0.803 | 3.27E-04 |
| Ryuvidine 4uM | H4K20me1 | 60 | 0.323 | 0.259 | 0.744 | 3.28E-14 |
| Ryuvidine 4uM | H3K27me3 | 60 | 2.272 | 2.126 | 1.487 | 6.74E-02 |
| Ryuvidine 4uM | MacroH2A | 61 | 3.135 | 2.781 | 2.036 | 1.05E-02 |
| Ryuvidine 4uM | ubH2A | 66 | 2.061 | 1.684 | 2.625 | 1.52E-08 |
| Ryuvidine 4uM | SMCHD1 | 60 | 2.366 | 2.093 | 1.321 | 2.69E-02 |
| GSK343 5uM | H4K20me1 | 60 | 3.011 | 2.555 | 2.250 | 2.12E-01 |
| PRT4165 50uM | H4K20me1 | 60 | 3.613 | 3.070 | 1.843 | 3.12E-04 |
| GSK343 5uM | H3K27ac | 60 | -2.031 | -1.875 | 0.844 | 2.05E-01 |
| PRT4165 50uM | H3K27ac | 60 | -2.370 | -2.151 | 0.966 | 7.23E-01 |

**Table S8: list of primers used throughout this experiment**

The names of the various primers used throughout the experiments described. Primers were labeled either F (forward) or R (reverse) to denote their orientation during amplification. All sequences are orientated 5’ to 3’.

| Primer name | Sequence (5’ to 3’) | notes |
| --- | --- | --- |
| qPCDNA5 F | GCTCGTTTAGTGAACCGTCAGA |  |
| qPCDNA5 R | GGTCCCGGTGTCTTCTATGGA |  |
| *PGK1* F | GCAGTCGGCTCCCTCGTT |  |
| *PGK1* R | AACGACCCGCTTCCCTTTA |  |
| *UBC* F | TGTCAAGGCAAAGATCCAAGATAA |  |
| *UBC* R | TCCAGCTGTTTTCCAGCAAA |  |
| *CTSB* F | TCGTGCACTCTGCTAATCATG |  |
| *CTSB* R | CAGTGGGTCAGAAACAACTCC | biotinylated |
| *CTSB* SNP | TTTACAGATTGCCTCCT |  |
| *DLC1* F | TTGATGGCTTCCAGATTTGTAA |  |
| *DLC1* R-M13 | CGCCAGGGTTTTCCCAGTCACGACAGCACAGTGGACATGTTTCTTAAT |  |
| *DLC1* SNP | GCTTCCAGATTTGTAAGATT |  |
| *SLC25A37* F | GATTTTCTTGAGGGCTCCGTAG |  |
| *SLC25A37* R-M13 | CGCCAGGGTTTTCCCAGTCACGACCAGATCCCAAAGCCCAGTACAC |  |
| *SLC25A37* SNP | TTGAGGGCTCCGTAG |  |
| *STC1* F | TTCATTTTAGGGGTGTTGACACA |  |
| *STC1* R | AAAAAAATCAAACCAGGCACAGT | biotinylated |
| *STC1* SNP | TAGGGGTGTTGACACACCA |  |
| M13 R | CAGGAAACAGCTATGAC | biotinylated |

**Table S9: Table of antibodies used for IF and western blotting**

| Name | Company | Catalog # | Host species |
| --- | --- | --- | --- |
| Anti-ubiquitin-Histone H2A Antibody | Sigma-Aldrich | 05-678 | mouse |
| Anti-H4 Antibody | Upstate | 25296 | rabbit |
| β-actin loading control antibody | Invitrogen | MA5-15739 | mouse |
| H3K27me3 Antibody | Diagenode | C15410069 | rabbit |
| CIZ1 Antibody (A-6) | Santa Cruz Biotechnology | sc-393021 | mouse |
| Anti-monomethyl-Histone H4 (Lys20) Antibody | Upstate | 07-440 | rabbit |
| Histone H3K27ac antibody | Active Motif | 39133 | rabbit |
| Anti-Histone Macro H2A Antibody | Upstate | 07-219 | rabbit |
| Anti-SMCHD1 antibody | Abcam | ab31865 | rabbit |
| Goat anti-Rabbit IgG (H+L) Cross-Adsorbed Secondary Antibody, Alexa Fluor 594 | Invitrogen | A11012 | goat |
| Goat anti-Mouse IgG (H+L) Cross-Adsorbed Secondary Antibody, Alexa Fluor 594 | Invitrogen | A11005 | goat |
| IRDye 680RD Goat anti-Rabbit IgG | Licor | 925-68071 | goat |
| IRDye 800CW Goat anti-Mouse IgG | Licor | 925-32210 | goat |
