# Supplementary figures and images for: Multiple distinct domains of human XIST are required to coordinate gene silencing and subsequent heterochromatin formation
Source: Epigenetics Chromatin. 2022 Feb 4;15:6. doi: 10.1186/s13072-022-00438-7 (PMC8815261; doi:10.1186/s13072-022-00438-7)

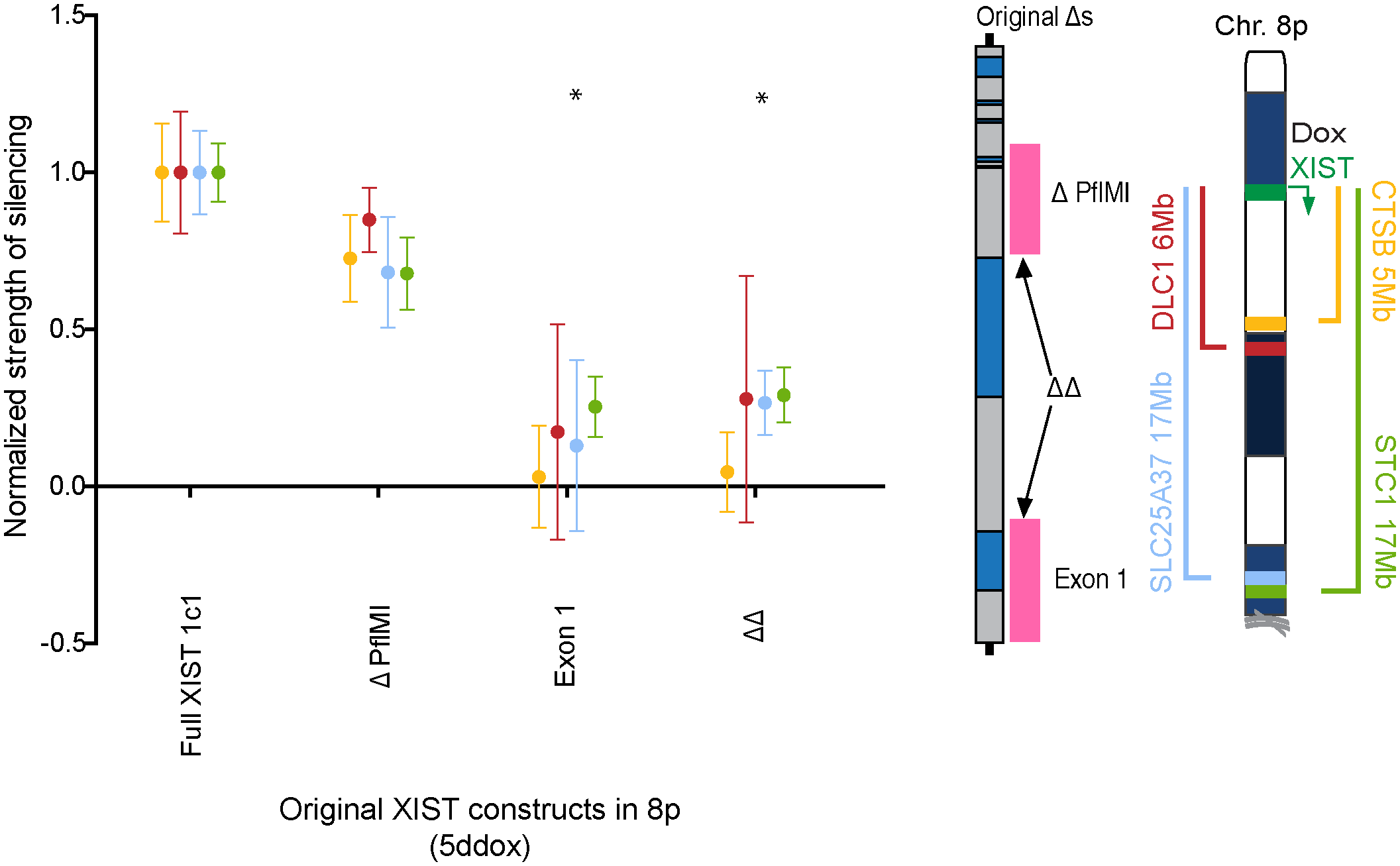

Supplement: Supplementary file 2 — Additional file 2: Fig. S1. Original XIST deletion constructs confirm importance of 3’ region for silencing of transcription. The ability of the original deletion constructs (Delta PflMI, Exon 1 and Delta Delta) to silence four distal genes. Strength of silencing was normalized to Full XIST. Three biological replicates were tested for each condition shown and statistical significance of a treatment’s effect on XIST induced silencing was calculated using a t-test with multiple testing correction and those constructs where silencing of all four genes were impacted are shown (*p < 0.05). [file 13072_2022_438_MOESM2_ESM.png]

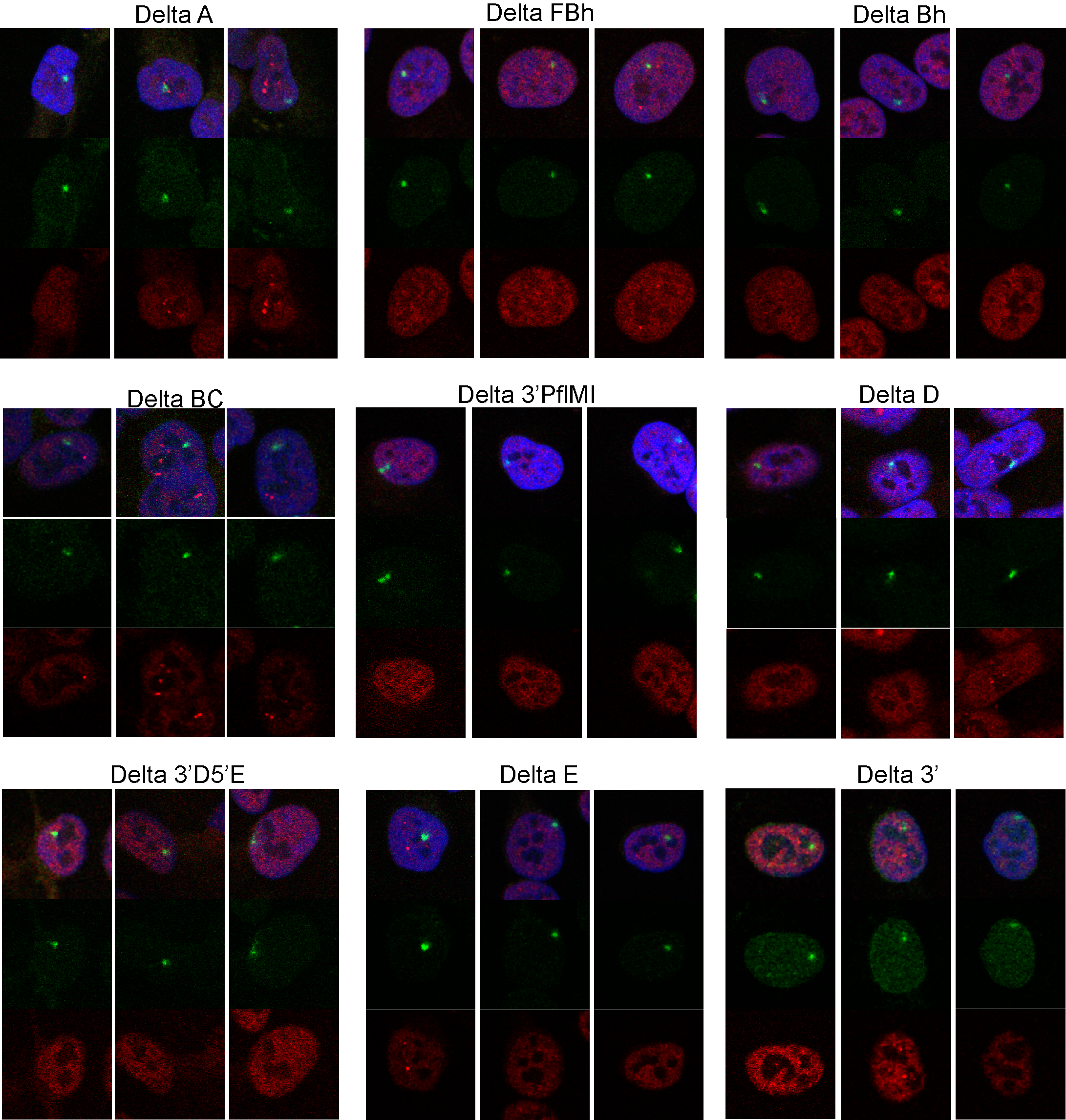

Supplement: Supplementary file 3 — Additional file 3: Fig. S2. Example images of Cot-1 depletion in XIST deletion constructs. Example IF-FISH images of the nuclei of HT1080 XIST CRISPR deletion constructs following 5 days of dox induction. The top three photos for each type of deletion construct are the merged colour channels showing DAPI (blue), XIST RNA (green) and Cot-1 (red). Below each merged channel is the single channel of XIST RNA (green) and Cot-1 red. All images are squares with each side ranging from 24 to 27 µm in length. [file 13072_2022_438_MOESM3_ESM.png]

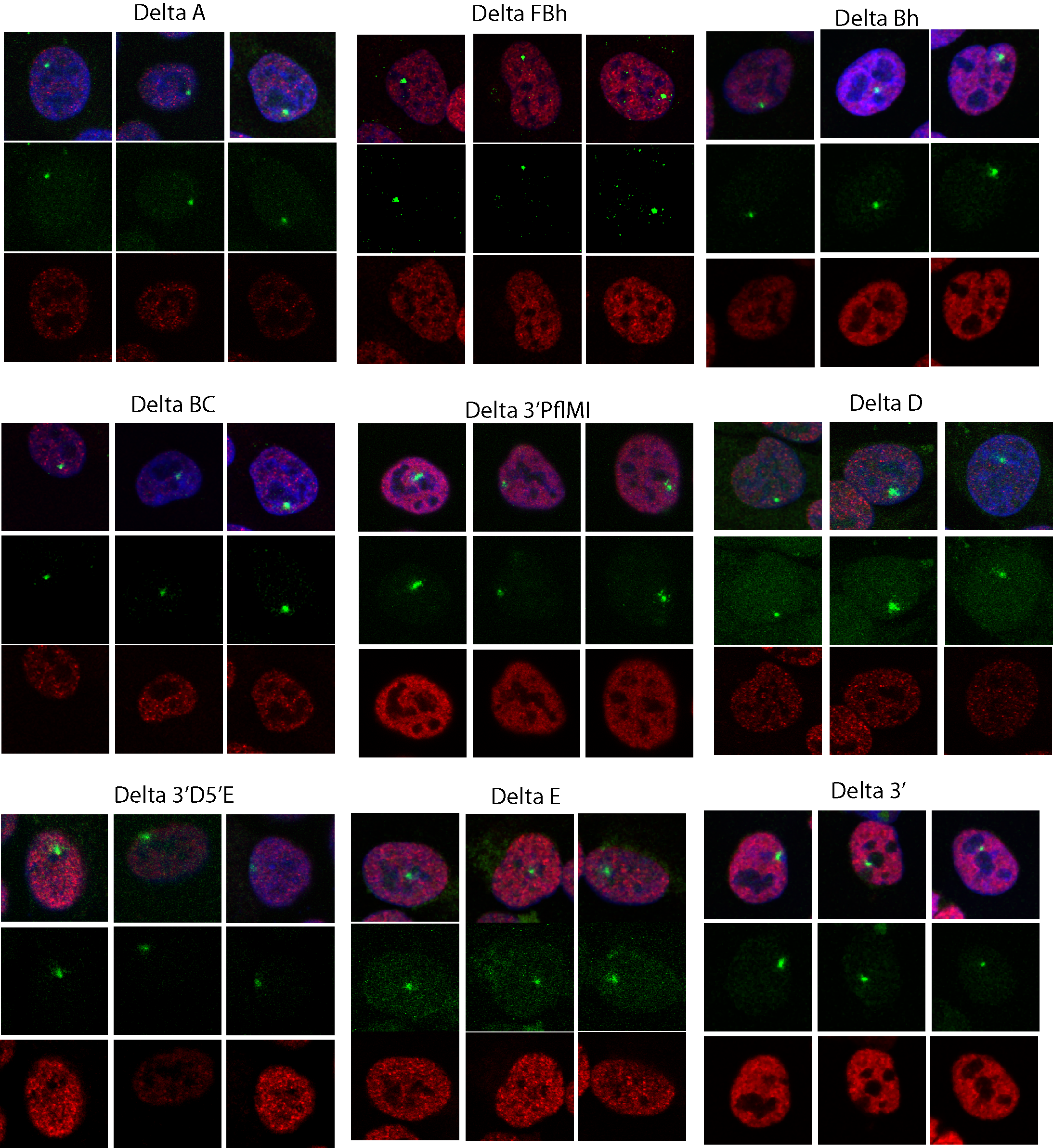

Supplement: Supplementary file 4 — Additional file 4: Fig. S3. Example images of H3K27ac depletion in XIST deletion constructs. Example IF-FISH images of the nuclei of HT1080 XIST CRISPR deletion constructs following 5 days of dox induction. The top three photos for each type of deletion construct are the merged colour channels showing DAPI (blue), XIST RNA (green) and H3K27ac (red). Below each merged channel is the single channel of XIST RNA (green) and H3K27ac (red). All images are squares with each side ranging from 24 to 27 µm in length. [file 13072_2022_438_MOESM4_ESM.png]

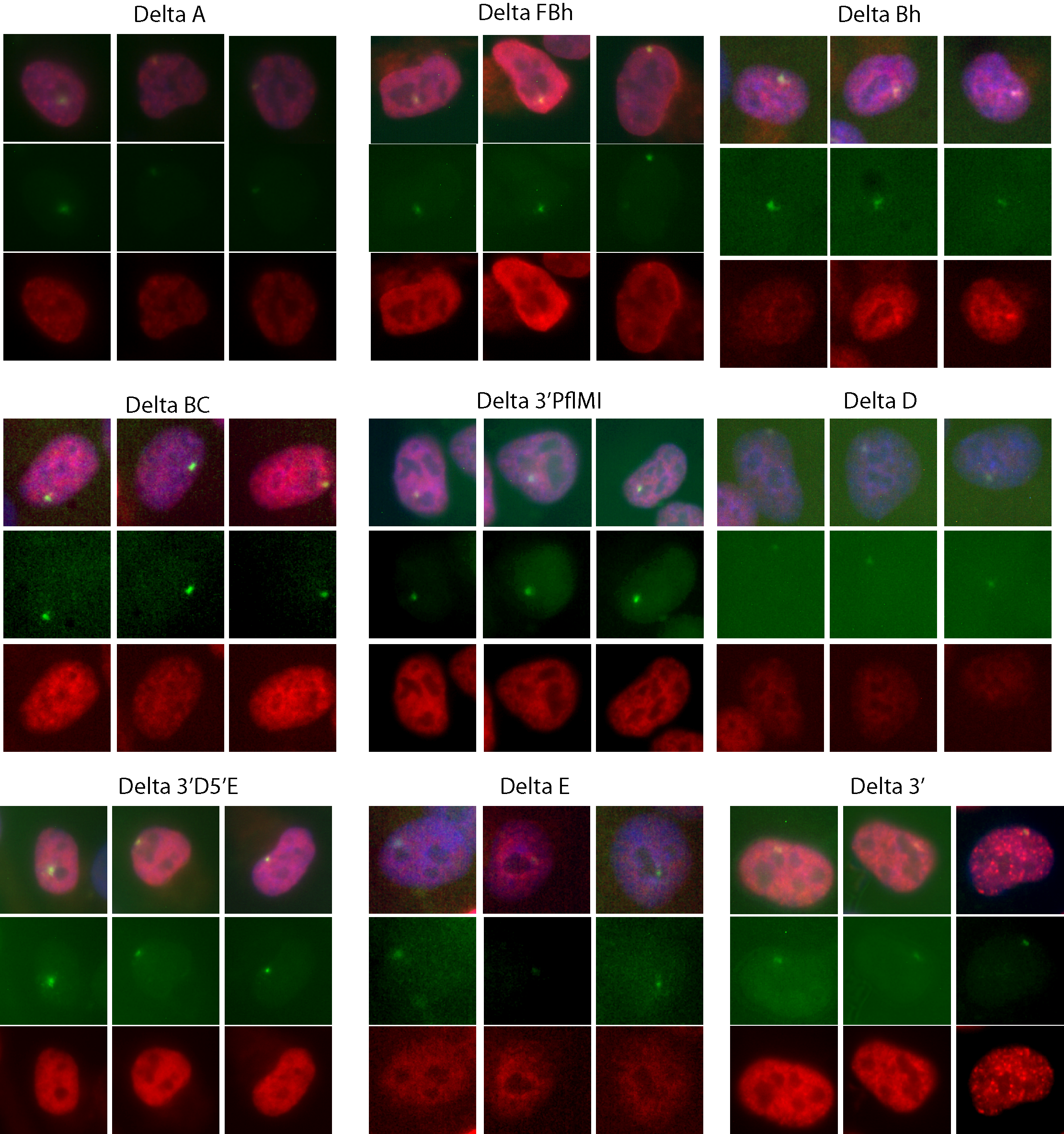

Supplement: Supplementary file 5 — Additional file 5: Fig. S4. Example images of H4K20me1 enrichment in XIST deletion constructs. Example IF-FISH images of the nuclei of HT1080 XIST CRISPR deletion constructs following 5 days of dox induction. The top three photos for each type of deletion construct are the merged colour channels showing DAPI (blue), XIST RNA (green) and H4K20me1 (red). Below each merged channel is the single channel of XIST RNA (green) and H4K20me1 (red). All images are squares with each side ranging from 24 to 27 µm in length. [file 13072_2022_438_MOESM5_ESM.png]

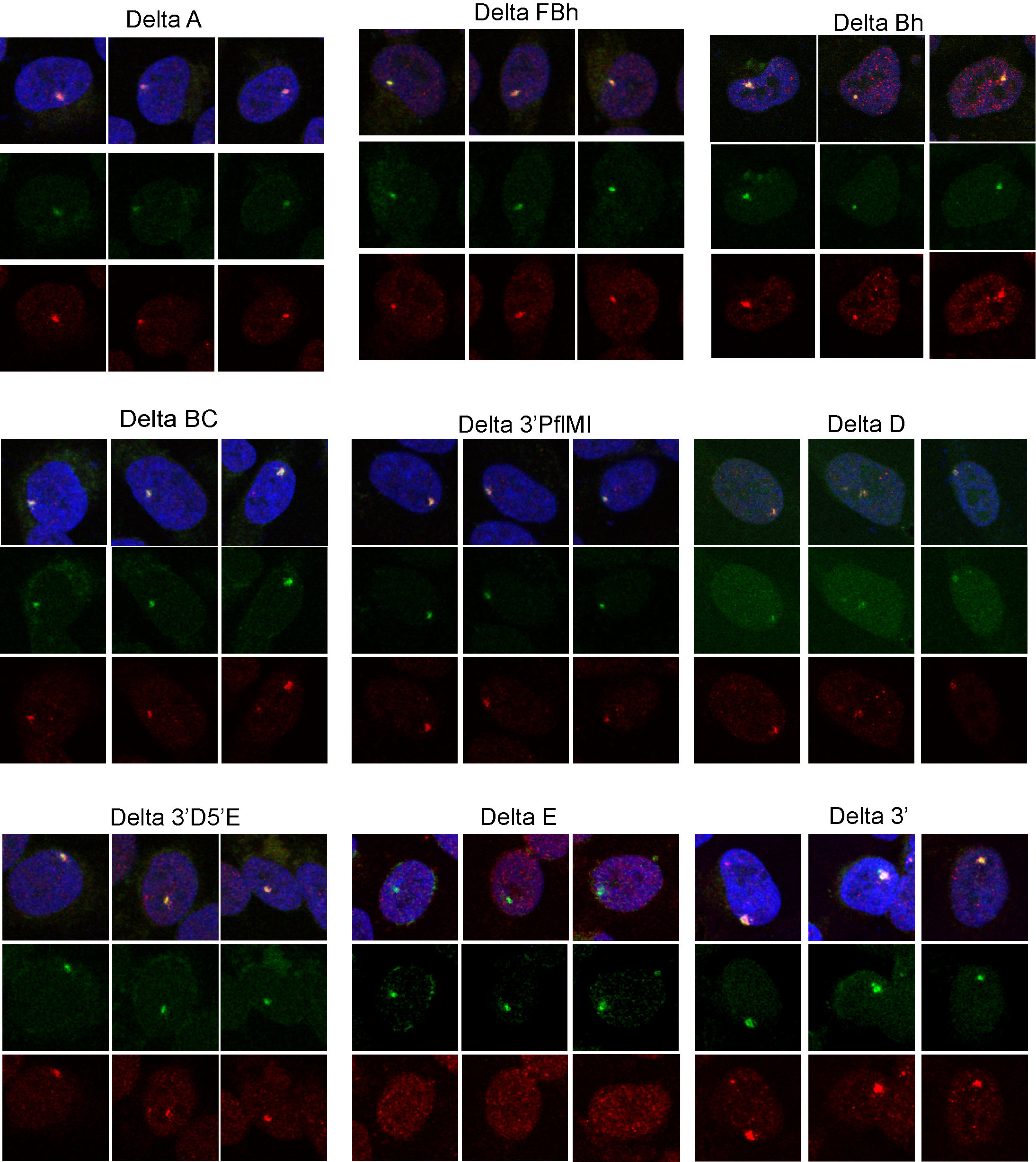

Supplement: Supplementary file 6 — Additional file 6: Fig. S5. Example images of CIZ1 depletion in XIST deletion constructs. Example IF-FISH images of the nuclei of HT1080 XIST CRISPR deletion constructs following 5 days of dox induction. The top three photos for each type of deletion construct are the merged colour channels showing DAPI (blue), XIST RNA (green) and CIZ1 (red). Below each merged channel is the single channel of XIST RNA (green) and CIZ1 (red). All images are squares with each side ranging from 24 to 27 µm in length. [file 13072_2022_438_MOESM6_ESM.png]

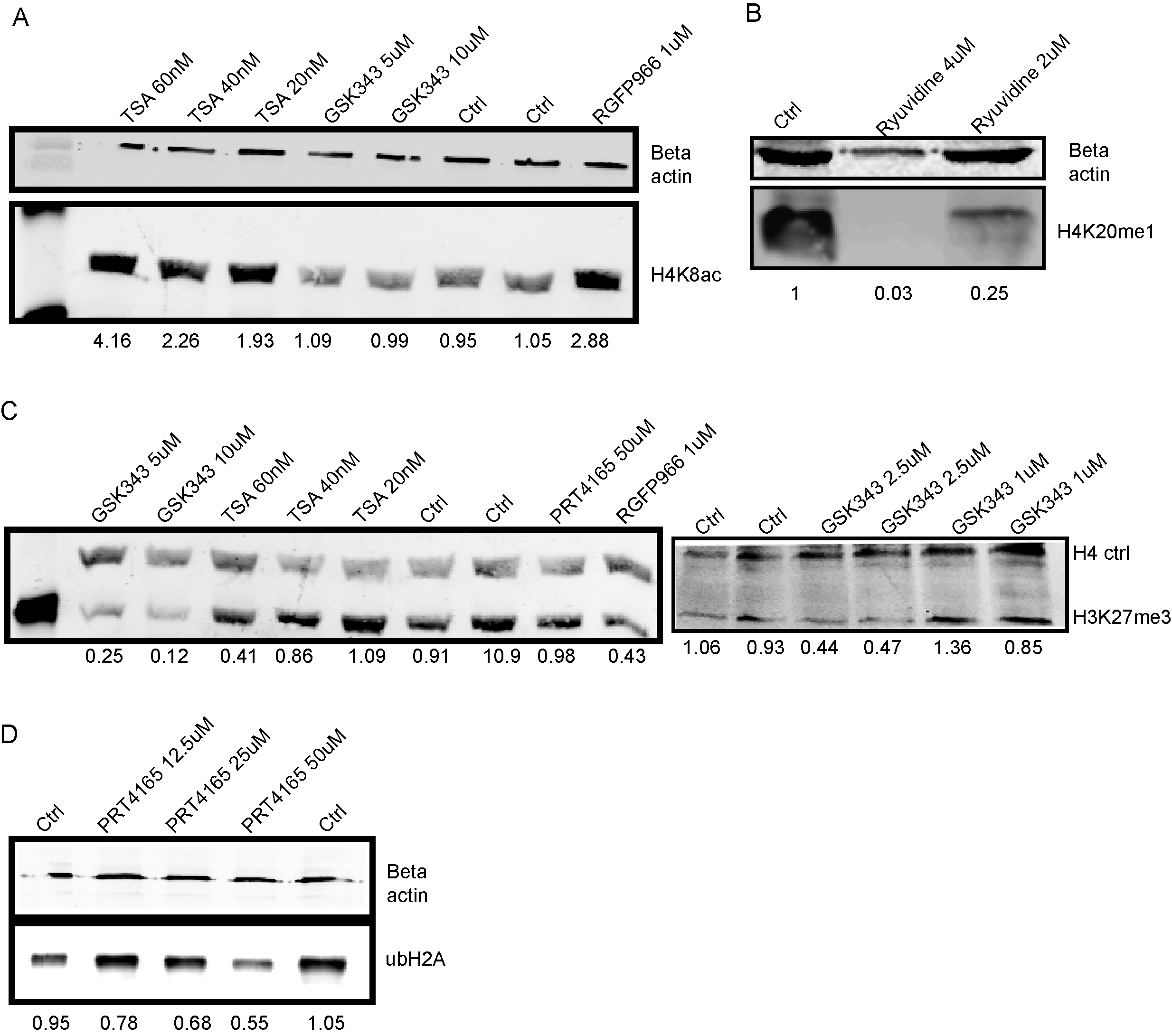

Supplement: Supplementary file 7 — Additional file 7: Fig. S6. Western blots showing effect of chemical inhibitors on global histone modification levels. Western blotting images demonstrating the levels of modified histone marks following treatment of HT1080 cells with chemical inhibitors. The bands for each chromatin feature as well as endogenous control are shown and the computed relative levels of each chromatin mark relative to the uninhibited controls are shown underneath the bands. A) Levels of histone acetylation (H4K8ac) following HDAC treatment as well as treatment with GSK343. B) Levels of H4K20me1 following Ryuvidine treatments C) Effect of GSK343 inhibition on H3K27me3 levels, in addition to HDAC inhibitors. D) Levels of ubH2A following PRC1 inhibition with PRT4165. C-D) Versions of these western blots were published previously in our investigation of the effect of GSK343 and PRT4165 on HT1080 cells [13]. The inhibitors used to target the activity of each factor are: TSA, broad spectrum HDACs; RGFP966, HDAC3; ryuvidine, PRSET7; GSK343, PRC2; PRT4165, PRC1. [file 13072_2022_438_MOESM7_ESM.png]

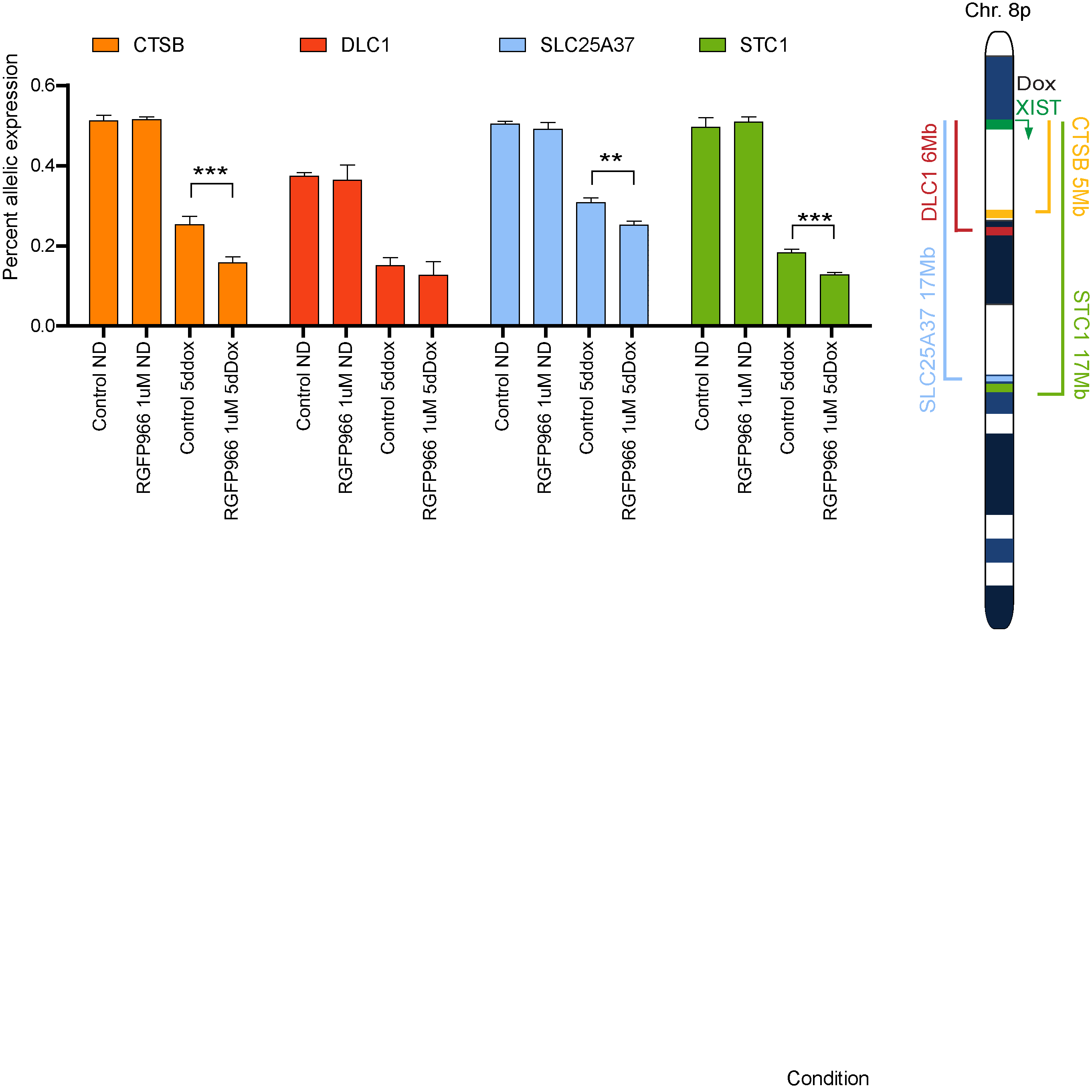

Supplement: Supplementary file 8 — Additional file 8: Fig. S7. Allelic contribution unaffected by HDAC3 inhibition in absence of XIST. The allelic contribution of the four SNP coding alleles is shown as measured by pyrosequencing. Allelic contribution to the expression of each gene is shown in the presence or absence of HDAC3 inhibitor, RGFP966, for five days with or without the induced expression of XIST with dox. Statistical significance was calculated using a t-test with thresholds adjusted for multiple testing correction from * < 0.05, ** < 0.01, *** < 0.001. [file 13072_2022_438_MOESM8_ESM.png]

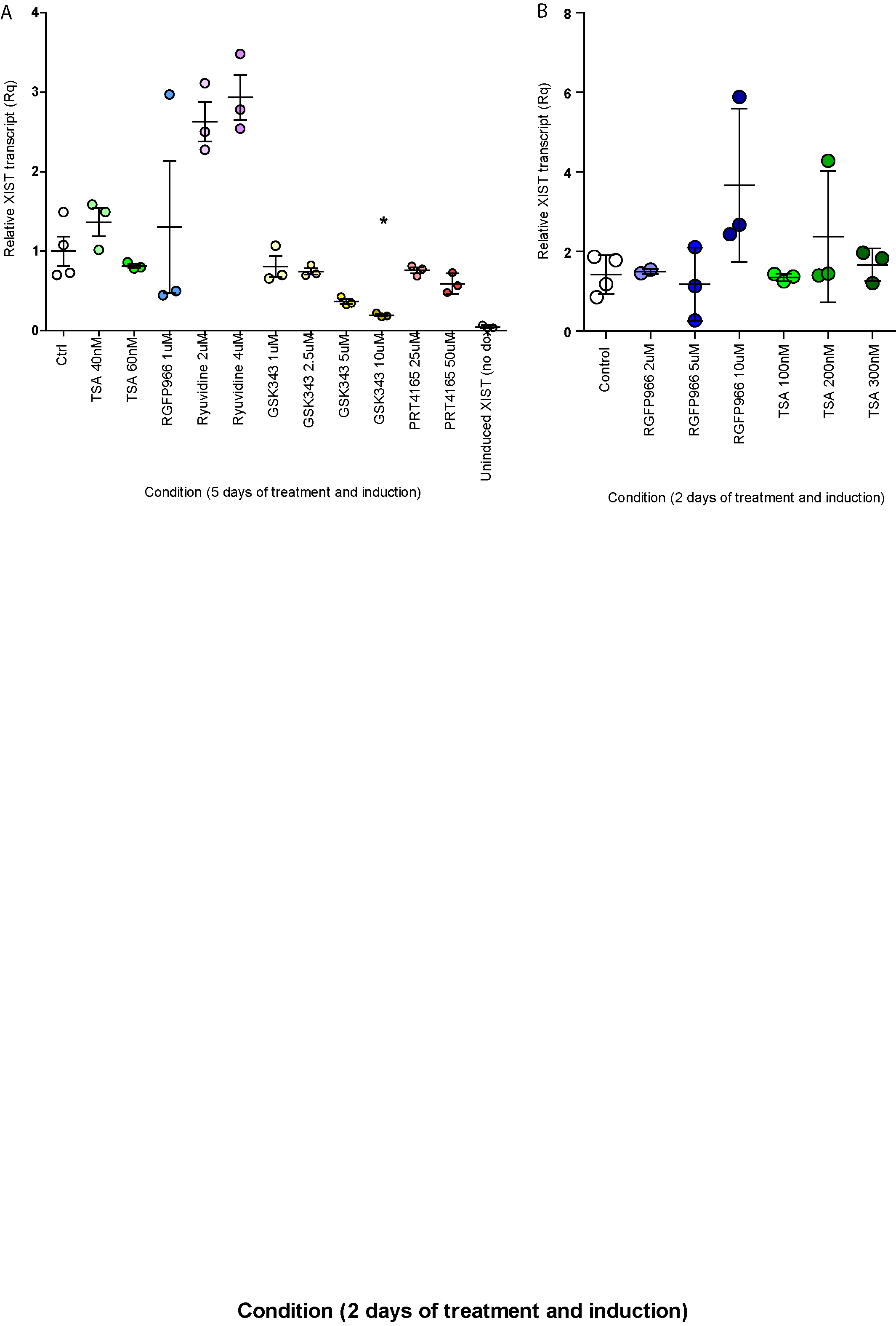

Supplement: Supplementary file 9 — Additional file 9: Fig. S8. Effect of chemical inhibitors on XIST RNA levels. Relative expression of XIST RNA following treatment of HT1080 cells with chemical inhibitors. Individual dots for each treatment condition show the relative levels of biological replicates compared to the average levels of time matched dox treated cells. Differences in XIST levels in any of the treatment conditions were calculated using t-test with multiple testing correction (*p < 0.05). A) Relative levels of XIST following five days of induction with 1ug/ml dox and the chemical inhibitor listed. B) Relative levels of XIST following two days of induction with 1ug/ml dox and the chemical inhibitor listed. [file 13072_2022_438_MOESM9_ESM.png]

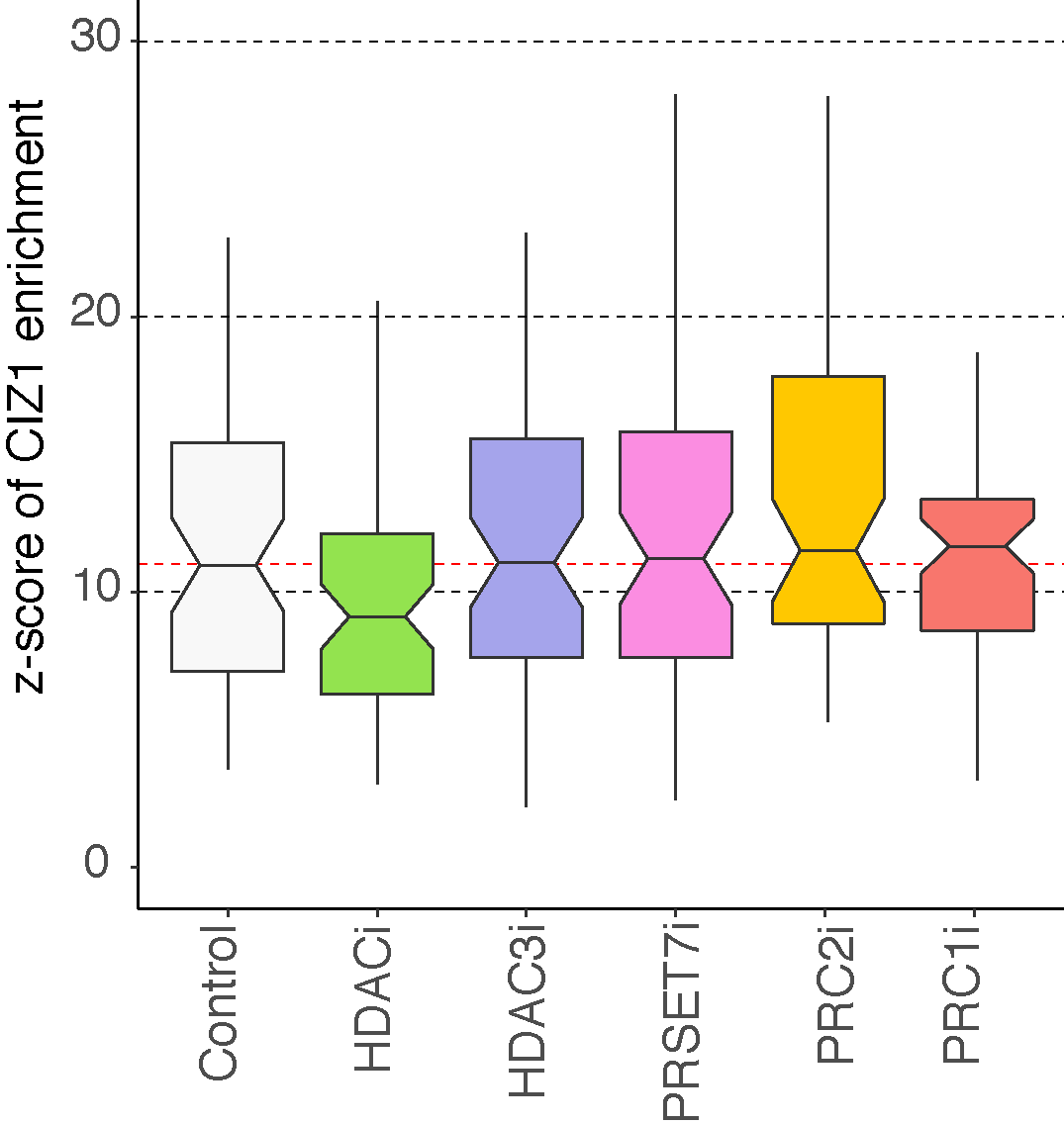

Supplement: Supplementary file 10 — Additional file 10: Fig. S9. Effect of chemical inhibition on the enrichment of CIZ1 at XIST RNA. The relative levels of CIZ1 (z-score) at the site of XIST RNA in the HT1080 cell lines following five days of chemical inhibition treatment. Populations of 60 cells were analyzed per condition and were compared by Mann–Whitney test to an uninhibited control population of HT1080 cells, all of which had Full XIST induced for 5 days. None of the conditions differed significantly from the control. [file 13072_2022_438_MOESM10_ESM.png]
